# Dual synergistic inhibition of COX and LOX by potential chemicals from Indian daily spices investigated through detailed computational studies

**DOI:** 10.1038/s41598-023-35161-0

**Published:** 2023-05-27

**Authors:** Mithun Rudrapal, Wafa Ali Eltayeb, Gourav Rakshit, Amr Ahmed El-Arabey, Johra Khan, Sahar M. Aldosari, Bader Alshehri, Mohnad Abdalla

**Affiliations:** 1 Department of Pharmaceutical Sciences, School of Biotechnology and Pharmaceutical Sciences, Vignan’s Foundation for Science, Technology & Research (Deemed to Be University), Guntur, 522213 India; 2 Biotechnology Department, Faculty of Science and Technology, Shendi University, Shendi, 414601 Sudan; 3 Department of Pharmaceutical Sciences and Technology, Birla Institute of Technology, Ranchi, 835215 India; 4 Department of Pharmacology and Toxicology, Faculty of Pharmacy, Al-Azhar University, Cairo, 11651 Egypt; 5 Department of Medical Laboratory Sciences, College of Applied Medical Sciences, Majmaah University, Al’Majmaah, 11952 Saudi Arabia; 6 Health and Basic Sciences Research Center, Majmaah University, Al’Majmaah, 11952 Saudi Arabia; 7 Pediatric Research Institute, Children’s Hospital Affiliated to Shandong University, Jinan, 250022 People’s Republic of China

**Keywords:** Computational biology and bioinformatics, Diseases, Health care, Chemistry

## Abstract

Cyclooxygenase (COX) and Lipoxygenase (LOX) are essential enzymes for arachidonic acid (AA) to eicosanoids conversion. These AA-derived eicosanoids are essential for initiating immunological responses, causing inflammation, and resolving inflammation. Dual COX/5-LOX inhibitors are believed to be promising novel anti-inflammatory agents. They inhibit the synthesis of prostaglandins (PGs) and leukotrienes (LTs), but have no effect on lipoxin formation. This mechanism of combined inhibition circumvents certain limitations for selective COX-2 inhibitors and spares the gastrointestinal mucosa. Natural products, i.e. spice chemicals and herbs, offer an excellent opportunity for drug discovery. They have proven anti-inflammatory properties. However, the potential of a molecule to be a lead/ drug candidate can be much more enhanced if it has the property of inhibition in a dual mechanism. Synergistic activity is always a better option than the molecule's normal biological activity. Herein, we have explored the dual COX/5-LOX inhibition property of the three major potent phytoconsituents (curcumin, capsaicin, and gingerol) from Indian spices using in silico tools and biophysical techniques in a quest to identify their probable inhibitory role as anti-inflammatory agents. Results revealed the dual COX/5-LOX inhibitory potential of curcumin. Gingerol and capsaicin also revealed favorable results as dual COX/5-LOX inhibitors. Our results are substantiated by target similarity studies, molecular docking, molecular dynamics, energy calculations, DFT, and QSAR studies. In experimental inhibitory (in vitro) studies, curcumin exhibited the best dual inhibitory activities against COX-1/2 and 5-LOX enzymes. Capsaicin and gingerol also showed inhibitory potential against both COX and LOX enzymes. In view of the anti-inflammatory potential these spice chemicals, this research could pave the way for more scientific exploration in this area for drug discovery.

## Introduction

Non-communicable diseases (NCDs) lie in the epicenter among all diseases as they represent the most prominent class in the death rate. The global chronic disease epidemic is responsible for the premature deaths of about 17 million people (80%) annually^1^. Reducing risk factors might be considered an approach to tackling such chronic illness^2^. As evidence links many chronic diseases to inflammatory events, modulation of the inflammatory cascade appears to be a critical new approach to preventing these conditions^3^. Inflammation may be caused by various agents such as pathogens (bacteria, viruses, or fungi), external body injuries, chemicals or radiation effects, and a diseased condition (cystitis, bronchitis, otitis media, dermatitis etc.). Severe inflammation, in some cases, might cause allergic reactions. Mostly all diseases are connected to inflammation, viz. arthritis (autoimmune disease), HIV (human immunodeficiency virus), psoriasis, TB (Tuberculosis), cancer, pneumonia, asthma, hypersensitivities, inflammatory bowel diseases, interstitial cystitis, vasculitis, chronic prostatitis, and many more^4^.

**Figure 1:**
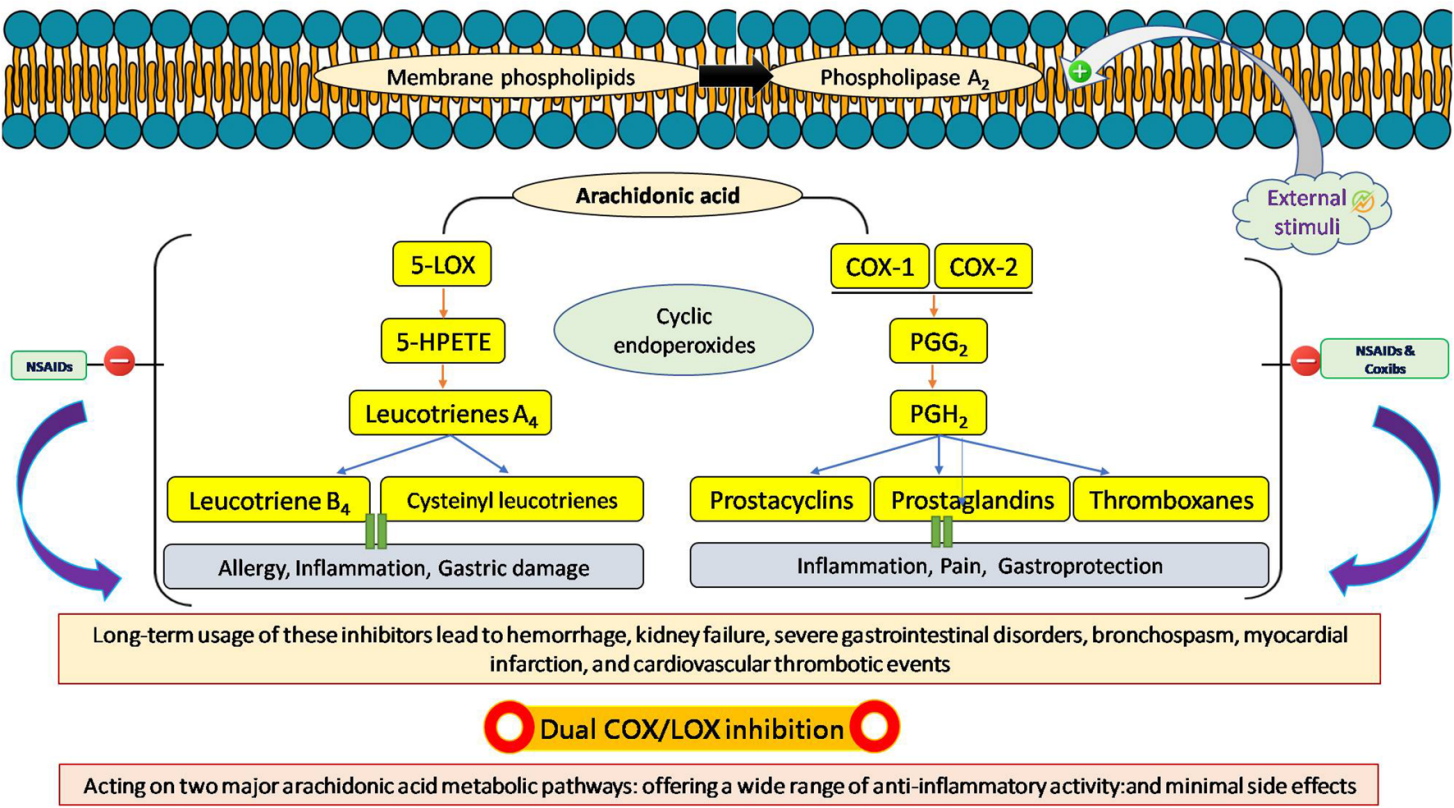
Concept of dual COX-1/2 and 5-LOX inhibition.

Many human diseases can be traced back to arachidonic acid cascade inflammatory mediators produced by the cyclooxygenase (COX) and lipoxygenase (LOX) pathways. Arachidonic acid (AA) is metabolized by a series of enzymes known collectively as cyclooxygenases (COX-1/2) and lipoxygenases (LOX)^5^. Eicosanoids, such as leukotrienes, prostaglandins, and thromboxane, are major inflammatory mediators, and their physiological production requires these enzymes.COX-1 is an essential enzyme that catalyzes the biosynthesis of eicosanoids which in turn promotes platelet aggregation, thromboxane, and vasoconstriction. It is mostly found in the kidney, stomach, and platelets^6^. Inhibition of this enzyme could help reduce biomarkers of inflammation. Non-steroidal anti-inflammatory drugs (NSAIDs), whether selective or non-selective, are commonly used as the first line of treatment in inflammatory diseases, because cyclooxygenase isoenzymes (COX-1 and COX-2) are responsible for the production of prostaglandins that play an important role in inflammation. They suppress the formation of pro-inflammatory eicosanoids. However, the long-term usage of these inhibitors may lead to bleeding, kidney failure, severe gastrointestinal disorders, and bronchospasm^7^. To avoid side effects, scientists had focused on developing selective COX-2 inhibitors in response to mounting evidence that this particular isoenzyme is over-expressed in inflammatory conditions, as opposed to COX-1 or combined COX-1/COX-2 inhibitors (for example, ibuprofen), which are associated with a wide range of unpleasant side effects^8^. Eicosanoids are released by the second essential biosynthetic pathway, activated by 5-lipoxygenase (5-LOX), an isozyme of LOX. Leukotriene B_4_, a mediator of atherosclerosis, cancer, and cardiovascular disease, is produced at the termination of the 5-LOX pathway. Thus, lowering leukotrienes by inhibiting 5-LOX may mitigate the risks of selective COX-2 and COX-1 inhibitors on the cardiovascular and gastrointestinal systems. With this perspective, it was anticipated that co-inhibition of cyclooxygenases (COX-I and COX-II) and 5-lipoxygenase (5-LOX) could lessen the cardiovascular and gastrointestinal side effects, while preserving the drugs' primary effectiveness against cyclooxygenase, particularly COX-2^9,10^. Dual COX/5-LOX inhibitors offer numerous therapeutic benefits over traditional NSAIDs; firstly, they act on the two major arachidonic acid metabolic pathways and possess a wide range of anti-inflammatory activity. Secondly, dual inhibitors provide enhanced gastric protection and a safer cardiovascular profile, which is COX inhibitor’s most cumbersome side effect^13^. As a result, there has been significant effort in the field of dual-acting COX/5-LOX inhibitors in recent years, with very promising results. Darbufelone and Licofelone were developed and tested in humans as COX/5-LOX inhibitors. High toxicity and low efficacy prevented their commercialization, though. Hence, the need is to discover or repurpose drugs that could have dual COX/5-LOX inhibitory nature as both COX and LOX derivatives (prostanoid and leukotrienes, respectively) are involved in other diseases than inflammation such as cancer proliferation wherein the use of dual inhibitors could be an interesting approach. In light of these ideas, it has been proposed that inhibiting the production of prostaglandins and leukotrienes could have synergistic effects and lead to the greatest reduction in inflammatory reactions possible^11,12^. Figure 1 displays the rationale behind finding a molecule with a dual inhibitory nature to widen the anti-inflammatory space. Further, the pathogenic processes of infectious diseases (bacterial or viral) or many other diseases are known to include inflammation, yet the existing treatments have undesirable side effects.

In this regard, phytomedicine offer an excellent source of bioactive compounds that could serve the purpose above. Their bioactive constituents or natural products are resonably safer and more active than synthetic compounds^13^. Numerous studies on plant whole crude extracts have been conducted over the past few decades, and it has been discovered that several plants have anti-inflammatory properties. Multiple inflammatory conditions can be effectively treated with compounds derived from plants. Alkaloids, flavonoids, polyphenols, and terpenoids are the few chemical classes linked to anti-inflammatory effects. Some well-known examples include andrograpanin, azadirachtin, Curcumin, embelin, resveratrol, and quercetin. Polyphenols are essential anti-inflammatory compounds found in spices that help improve health and decrease undesirable medication reactions. The discovery of flavonoids as a valuable substitute for traditional analgesics and anti-inflammatory agents with dual-inhibitory action, especially on COX-2 and 5-LOX, can reduce or resolve this issue and has attracted much attention. The anti-inflammatory compound flavocoxid, which contains the flavonoids catechin and baicalin, works by simultaneously inhibiting the COX 1/2 and 5-LOX enzymes^14^. Flavonoids are potent antioxidants that may regulate the oxidative generation of arachidonic acid from phospholipids, hence decreasing lipid peroxidation processes and activation of inflammatory pathways^15^. Flavocoxid suppresses inflammation not only by inhibiting cyclooxygenase (COX 1/2) and lipoxygenase (5-LOX), but also by lowering NF-B-induced production of pro-inflammatory cytokines^16^.

Many natural products have shown proven efficacy towards preventing/curing inflammation associated with various diseases in clinical investigations. Turmeric and its curcumin-enriched preparations have been used to treat arthritis and alleviate its symptoms i.e. morning stiffness of joints^17^. Ginger and its phenolic compounds (gingerdiol, gingerol, gingerdione and shogaols), sesquiterpenes have shown potent activity in reducing lung inflammation^18,19^. It is highly effective in inflammations associated with alimentary channel such as colitis and inflammatory bowel disease^20^. It also has clinical evidence in reducing the pain associated with osteoarthritis^21^. Polyphenols in green tea have anti-inflammatory effects. Green tea has been found in clinical research to lower inflammation related with cardiovascular disease, arthritis, and other inflammatory disorders^22^. It has also been demonstrated to be useful in lowering obesity-related inflammation and Alzheimer’s disease^23^. Capsaicin has anti-inflammatory activities^24^. In addition to these there are many other natural compounds which have clinically proven anti-inflammatory effects i.e. colchicine, resveratrol, epigallocatechin-3-gallate (EGCG), and quercetin^25^.

This shifts our attention to the common culinary spices, as since time immemorial, they have played a very significant role due to their traditional importance in fields like Ayurveda and homeopathy^26–30^. Medical practitioners have used them to treat various ailments, including inflammation^31^. As per reports, culinary herbs and spices exert anti-inflammatory activities by activating PPARα and PPARγ, thereby causing inhibition the activation of NF-κB and enhancing the expression of anti-inflammatory cytokines^32^. Studies on animal inflammation models have demonstrated that curcumin and capsaicin delay the development and minimize the incidence and severity of arthritis by inhibiting TNF-α, IL-1β, IL-6, and NF-κB (inflammatory cytokines)^33^. Curcumin is superior to aspirin in this situation because it selectively inhibits the development of the anti-inflammatory TXA_2_ without side effects. Capsaicin down regulates the expression of the COX-2 enzyme and represses the inducible nitric oxide synthase (iNOS) to reduce PGE2 synthesis. It suppresses the synthesis of pro-inflammatory mediators, TNF-α and IL-6, following the elevation in the production of anti-inflammatory mediator IL-10^34^. It has been demonstrated that capsaicin modulates the NF-B signaling pathway bythe formation of pro-inflammatory cytokines. It exerts an anti-inflammatory effect by blocking the signaling path, coupling pro-inflammatory stimuli with cyclooxygenase activation, including inhibiting NF-κB nuclear translocation^35^. Gingerol, the bioactive constituent of ginger, is anti-inflammatory, as it inhibits PGE_2_ generation and reduces nitric oxide production. They ultimately activate the endogenous antioxidant defense system by enhancing the generation of anti-inflammatory cytokines and inhibiting the formation of pro-inflammatory cytokines^36^. According to the meta-analysis, ginger consumption reduces C-reactive protein (CRP), high sensitivity C-reactive protein (hs-CRP), and tumor necrosis factor(TNF-α) considerably^37^. A wide array of spices and their phytoconstituents remain unexplored for their pharmacological activity due to limited studies.

In order to explore the role of phytoconstituents of common culinary spices in preventing/curing inflammation associated with various diseases, herein we aim to examine the role of culinary spice phytochemicals like curcumin, gingerol, and capsaicin from turmeric (*Curcuma longa*), ginger (*Zingiber officinale*) and red pepper (*Capsicum annuum*) and identifying the possible mechanistic pathway (COX/5-LOX inhibition) through in silico and in vitro studies^38,39^. The need for new anti-inflammatory agents having dual mechanisms with improved safety profiles that prevent the release of both prostaglandins and leukotrienes encouraged us to repurpose a few anti-inflammatory agents (curcumin, gingerol, and capsaicin) for their dual inhibitory activity to reclassify them as potent anti-inflammatory agents against both targets with a different probable mechanism of action. Their dual inhibitory activity and binding modes have been investigated using in silico tools^40,41^. Such a viewpoint opens the door, identifying culinary spices and herbs as a potential source of potent phytochemicals of the COX-1/2 and 5-LOX dual-route and offering it as an option in inflammation associated with various diseases. In view of the anti-inflammatory potential these spice chemicals, this study could pave a path for the scientific community to explore further in this direction, ultimately benefiting humanity.

## Materials and methods

### Target similarity

Target similarity represents the first step towards identifying dual inhibitors for any class. It helps identify structurally/functionally similar regions within proteins, which can justify it’s common evolutionary descent. Further, it supports the concept of dual inhibition by predicting the location and function of protein-coding and transcription-regulation regions in the genomic DNA by identifying conserved regions^42^. In this study, we have used a target-similarity approach to forecast the activity of a few natural products of flavonoid origin that would be reclassified as anti-inflammatory agents if they were found to exhibit activity against varied proteins related to anti-inflammatory pathways. This is based on the concept that a drug would behave similarly to its putative target if administered to a protein identical to it. The conserved amino acid residues for each protein relative to it’s homologous putative drug target were determined. It is considered that amino acid residues in proteins that have undergone evolutionary conservation play significant structural and functional roles. The pharmacological target was used as the subject, and its matching homolog as the query sequence in a protein–protein pairwise alignment using BLAST at National Center for Biotechnology Information (NCBI)^43^.

### Hardware and software employed

All simulation studies were performed on a DELL workstation running Ubuntu 20.04.3 LTS (64-bit as OS, Intel^®^ Core™ i7-11,800 CPU@2.30 GHz processor, 16 GB RAM). All co-crystallized protein structures were obtained from the Protein Data Bank (PDB) database^44^. Various modules of Schr $\ddot{o}$ dinger software were employed to perform molecular docking and molecular dynamics studies. Density functional theory (DFT) analyses were carried out using the Gaussian09 program^45^. The QSAR study was carried out usingHyperChem Professional 8.0.3 program.

### Molecular docking

#### Protein preparation

Three pro-inflammatory receptors/proteinswereemployed in this study. These are COX-1 (PDB ID:3N8Y)^46^, COX-2 (PDB ID:1CVU)^47^, and 5-LOX(PDB ID:3V99)^48^. The X-ray crystal structures of all proteins were downloaded from the Protein Data Bank^49^. The proteins were in complex with an inhibitor (co-crystallized/native ligand). The protein was prepared using the protein preparation wizard module of Schrödinger software^50^. The steps were addition of missing polar hydrogens, deletion of water molecules (beyond 5 Å) from hetero groups, ionization,generation of tautomeric statesat pH of 7.4 (per empirical pKa prediction), optimization of hydrogen bonds [predicted by PROPKA (pKa prediction)], and finally energy minimization.

#### Ligand preparation

Three phytocompounds, viz., curcumin, capsaicin, and gingerol were used as ligands for screening against COX-1, COX-2, and 5-LOX to determine their triple inhibitory potential. These ligand molecules are shown in Table 1 with their details. The ligand preparation steps involved (i) sketching individual structures in MarvinSketch 20.10 software, (ii) validating the correctness of each structure using the “structure checker” module of MarvinSketch 20.10 software, (iii) generation of 3D structures with Maestro12.3 module of Schrodinger software, (iv) optimization of structures using the LigPrep module (OPLS_2005 force field). Compound tautomers were disregarded, and instead, a single stereoisomer retaining desired chirality was generated for each compound.

**Table 1:** Phytomolecules employed as ligands with their code, structure, and details.

Sl. No.	Compound	Structure	Details
1	Curcumin		IUPAC: (1E,6E)-1,7-bis(4-hydroxy-3-methoxyphenyl)hepta-1,6-diene-3,5-dione Chemical formula: C_21_H_20_O_6_ Molecular weight: 368.39
2	Capsaicin		IUPAC: (E)-N-(4-hydroxy-3-methoxybenzyl)-8-methylnon-6-enamide Chemical formula: C_18_H_27_NO_3_ Molecular weight: 305.42
3	Gingerol		IUPAC: (S)-5-hydroxy-1-(4-hydroxy-3-methoxyphenyl)decan-3-one Chemical formula: C_17_H_26_O_4_ Molecular weight: 294.39

#### Protein–ligand docking

All molecular docking simulation studies were performed using the Ligand docking program of the Maestro 12.3 module of Schrodinger. Before the initiation of docking, the active site coordinates on the target proteins were generated employing the Receptor Grid Generation module. The van der Waals radii scaling factor and partial charge cutoff were kept at 1.0 and 0.25. The remaining parameters were kept as default. The Extra Precision (XP) mode was used to perform molecular docking of the three phytocompounds in the active site of three respective proteins. The binding affinities and molecular interactions were analyzed, and data were recorded. Lastly, the protein–ligand complex interactions were visualized in 2D/3D, and images were saved for representation.

### ADMET prediction

The pharmacokinetic process known as absorption, distribution, metabolism, and excretion (ADME) describes how the body responds to a drug. Predictive in silico ADME data aid the drug development process since it aids lead optimization. Several software programs were used to forecast the in silico ADMET attributes for all the molecules. In this study, the Swiss ADME, developed and maintained by the Swiss Institute of Bioinformatics (SIB) (https://www.swissadme.ch) webserver, wasemployed to compute the ADME values^51^. Predicting the in silico toxicity of a substance is essential for evaluating its safety profile. They not only aid in reducing the number of animal testing, but also help determine the maximum tolerable levels in animals. In this study, pkCSM web server (https://biosig.lab.uq.edu.au/pkcsm/prediction) was used to predict the pharmacokinetic properties/toxicities of our small molecules using graph-based signatures^52^. This server database provides various toxicity parameters, i.e. AMES toxicity, maximum tolerated dose, hepatotoxicity, skin sensitization, and hERG I and II inhibition. In both servers, the first step involves either uploading or writing the SMILES string of the particular molecule in the given space. The second step is to allow the server to run the program. Following this, the predictive data can be gathered and analyzed.

### Molecular dynamics simulation study

Molecular dynamics simulation (MDS) helps study the protein–ligand complex’s structural stability and flexibility. In this study, MDS was performed for the top hit compounds to legitimize the protein–ligand complex (PLC) and measure the ligand-binding constancy in the active site of the selected target^53,54^. MDS was carried out using the Desmond module of the Schrödinger Suite developed by the D.E. Shaw research group (Academic license)^55^. The system builder panel was used to build the orthorhombic simulation box with Simple Point-Charge (SPC)^56^. The periodic boundary conditions were set at 10 Å from the outer part of the protein surface. The TIP3P water model was used to solvate the system. To the solvated system, sufficient counter ions were added to neutralize the system. The 0.15 M NaCl was added to the simulation panel maintained an isosmotic state. OPLS_AA force field was applied to the protein–ligand complex. Energy minimization was carried out till the system reached a stable condition (1000 steps of steepest descent; conjugate gradient algorithm). This equilibrated system was used for the final production of the MD simulation for 100 ns at 310.15 K temperatures at 1.0 bar pressure with NPT (Isothermal-Isobaric ensemble^57^, i.e. constant temperature, constant pressure, constant number of particles) ensemble while using default settings for relaxation before simulation. The results of the MD simulation were analyzed by generating a simulation interaction diagram. RMSD, RMSF, protein–ligand interaction diagram, interacting amino acid residues with the ligand in each trajectory frame, and the trajectory of different ligand properties were analyzed.

### Molecular mechanics with generalized born and surface area solvation (MM-GBSA)

Post-simulation MM-GBSA analysis was carried out. The *thermal_MMGBSA.py script* of the Prime/Desmond module of the Schrödinger suite was used. The post-simulation MM-GBSA analysis of free binding energy calculation was carried out with the generation of 0–1000 frames. Two hundred frames were processed and analyzed throughout the MM-GBSA calculation of 100 ns MDS data. The binding free energies (kcal/mol) were then calculated.

### Density functional theory (DFT) calculations

DFT calculations help to investigate the electronic/nuclear structure of various systems. Based on quantum mechanical concepts, DFT computations enable predicting and estimating the system’s behavior^58^. Following the optimization, analysis was done using the Gauss View 6.0.16 program (Gaussian Inc., Wallingford, CT, USA, 2019).As one of the most reliable theoretical methods, density functional computations were used to conduct DFT experiments using the Gaussian09 suit programs^45^. In this study, calculations were performed using B3LYP and exchange–correlation functional with a basic set of 6-31G (d, p) for the atoms of carbon, nitrogen, oxygen, and hydrogen, and a theoretical geometry was established. For the ligands under study, DFT/B3LYP 6–31 + G (d, p) basis sets were used to assess single-point energy and dipole moment (D) values^59–61^. Calculated variables included the ionization energy, electron affinity, electronegativity, electronic chemical energy, chemical hardness, chemical softness, and electrophilicity index. Using DFT analysis, the energy difference between the highest occupied molecular orbital (HOMO) and lowest unoccupied molecular orbital (LUMO) energies were used to study the interactions of the ligand/s with the proteins^62^. The study considered the active site of the protein and examined the binding energies of the interacting residues.

### Quantitative structure-activity relationship (QSAR) study

The QSAR study was carried out using HyperChem Professional 8.0.3 program. The ligands were optimized by applying force field (MMþ) using semi-empirical PM3 methods. Energy minimization was carried out using the Fletcher–Reeves conjugate gradient algorithm. In addition, various other parameters like volume, hydration energy, Log P, refractivity, polarizability, mass, total energy, dipole moment, and RMS gradient were also computed for all the ligands under study.

### Experimental inhibition assays

#### In vitro cyclooxygenase (COX-1 and COX-2) inhibition assay

The inhibitory activities of the test compounds against COX-1 and COX-2 enzymes were determined by enzyme immune assay (EIA) kits (Cayman Chemical, Ann Arbour, MI, USA) using COX-1 (ovine) and COX-2 (human recombinant) according to reported methods^63–65^. The inhibitory activity was measured and reported as IC_50_ value (µM). Data are expressed as IC_50_ ± SEM for triplicate studies.

#### In vitro lipoxygenase (5-LOX) inhibition assay

The ability of the test compounds to inhibit the 5-LOX enzyme was evaluated using the Human Lipoxygenase Inhibitor screening assay (EIA) kit (Cayman Chemical). The IC_50_ values were measured in µM according to the manufacturer’s instructions and reported method^63–65^. Data are expressed as IC_50_ ± SEM for triplicate studies.

## Results

### Target similarity analysis

For dual inhibition of COX-1/2 and 5-LOX, all the major proteins involved in the inflammation pathways were selected based on literature review as we searched for significant proteins that influence inflammation. Keeping in pace with the concept of dual inhibition, three targets (COX-1/2 and 5-LOX) were selected,as presented in Table 2.

**Table 2:** Details of three targets selected in the study.

PDB ID	3N8Y	1CVU	3V99
Classification	Oxidoreductase		
Organism	*Ovis aries*	*Mus musculus*	*Homo sapiens*
Number of mutation	0	2	10
Co-crystallized ligand	2-[2,6-dichlorophenyl) amino] benzeneacetic acid, Diclofenac	Arachidonic acid	Arachidonic acid

**Figure 2:**
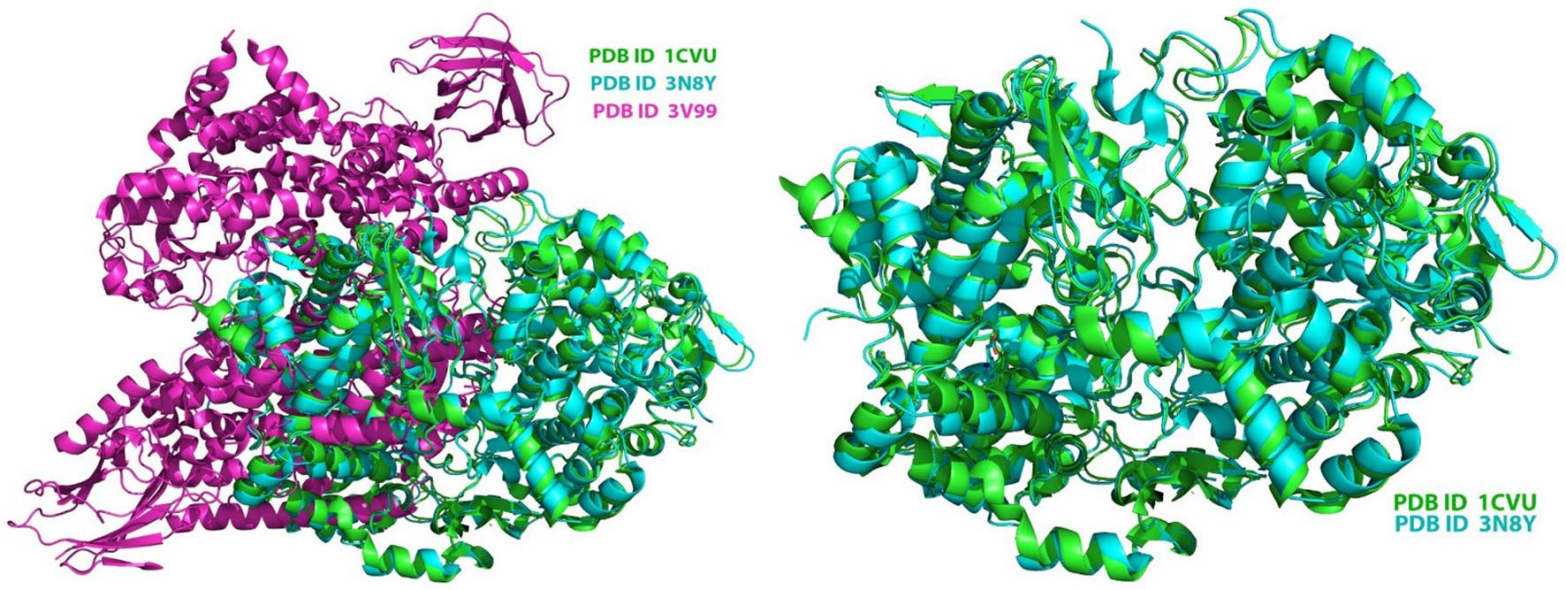
Structure comparison showing overlapping sequences of the amino acid residues for the proteins COX-1 (3N8Y), COX-2 (1CVU), and 5-LOX (3V99). COX-1: blue, COX-2: green, 5-LOX: magenta.

Sequence alignment was done for all three targets under study. Figure 2 highlights the sequence similarity for COX-1, COX-2, and 5-LOX showing maximum similarity.

**Figure 3:**
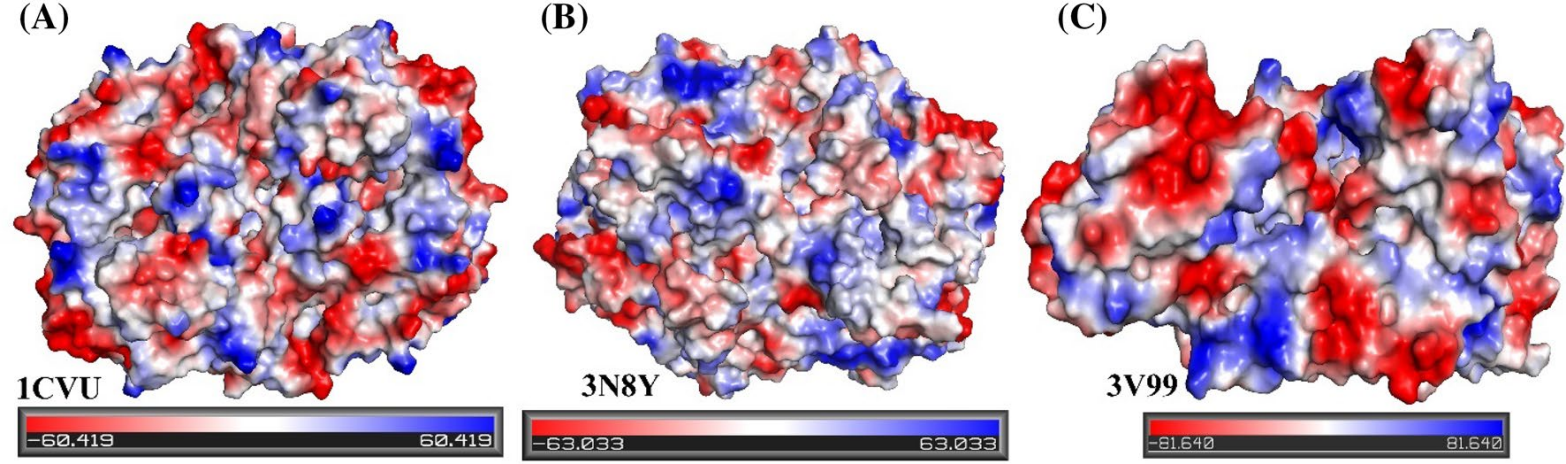
Electrostatic surface potential images of (**A**) COX-1 (3N8Y), (**B**) COX-2 (1CVU), and (**C**) 5-LOX (3V99) showing electropositive regions (colored blue) that may serve as the site of binding for various ligands.

To validate the results of target similarity, it is very important to locate the binding sites on the proteins to access the ligand interaction. For this, we evaluated the electrostatic surface potential (ESP) of the three studied proteins, as shown in Fig. 3^66^. The presence of multiple electropositive micro-domains being close to each other could favor the binding of small molecule inhibitors. The active site pocket of the COX-2 binding site is 20% larger than that of COX-1, whereas, in the case of 5-LOX,the active site is fully encapsulated. In the latter case, the amino acid residue Phe177plays a critical role and helps in the substrate/inhibitor binding in the active site^67^.

Further, His600 helps in substrate catalysis. Hence, the structural motif of 5-LOX is termed a “twist-and-pour cap”. Thus, from the above images and discussion, it can be interpreted that these three proteins share a high degree of similarity. Hence for our study, these three targets represent the primary class of proteins involved in the inflammatory pathways. This also justifies the rationale behind the design of dual inhibitors targeting COX-1/2 and 5-LOX.

### Molecular docking studies

#### Validation of docking procedure

**Figure 4:**
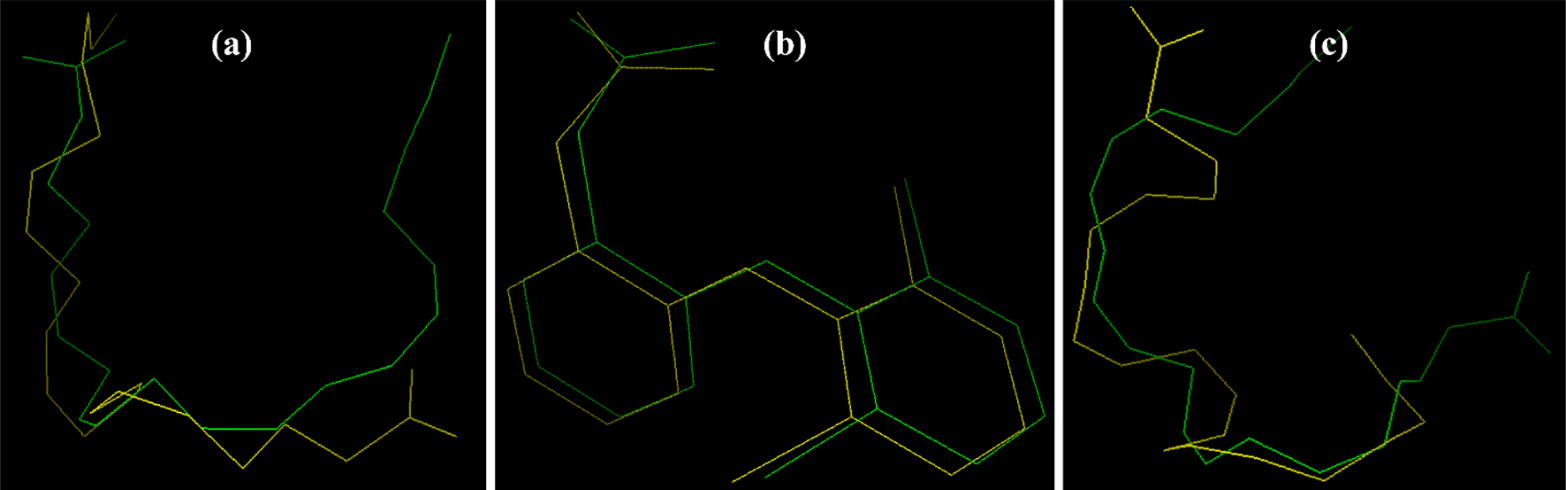
Docked internal ligand’s superimposed overlay conformation (**a**) ACD in relation to its crystallized conformation determined from the co-crystallized complex structure (PDB ID: 1CVU, (**b**) DIF in relation to its crystallized conformation determined from the co-crystallized complex structure (PDB ID: 3N8Y), and (**c**) ACD in relation to its crystallized conformation determined from the co-crystallized complex structure (PDB ID: 3V99).

The validation/re-docking studies (Fig. 4) on the crystal structure of COX-1and COX-2, and 5-LOXrevealed the binding energy of − 8.0 kcal/mol, − 8.5 kcal/mol, and − 6.1 kcal/mol, respectively with reference RMSD of 0.54 Å, 0.98 Å, and 1.24 Å. These minor RMSD fluctuations are acceptable for small molecules (0–3 Å)^55,68^.

#### Molecular docking simulation

The ligand docking study with COX-1/2 and 5-LOX enzymes revealed favorable binding energies. The binding affinities/docking scores of all molecules under investigation have been presented in Table 3. Further, as an observation, it was seen that only curcumin showed a better binding affinity than the reference ligand for all three proteins; this shows how well curcumin fits into the sub-pockets of COX-1/2 and 5-LOX enzymes. It can be interpreted from the docking scores that among all ligands, curcumin fits the best in the active site of all three proteins. Curcumin revealed a binding energy of − 9.2 kcal/mol and − 6.3 kcal/mol with COX-2 and 5-LOX, respectively. This binding energy was relatively high compared to the internal ligand’s binding state (− 8.0 kcal/mol and − 6.1 kcal/mol, respectively). However, in the case of COX-1, curcumin revealed a comparable binding affinity (− 6.7 kcal/mol) with the internal ligand (− 8.5 kcal/mol). The other ligands, capsaicin and gingerol showed comparable binding affinities towards all the receptors/enzymes compared to the internal ligand.

**Table 3:** Results of molecular docking study of curcumin, capsaicin, and gingerol against COX-1, COX-2, and 5-LOX.

Proteins(PDB ID)	Binding affinity/Docking score of ligands (kcal/mol)			
	Co-crystallized ligand	Curcumin	Capsaicin	Gingerol
3N8Y	− 8.5	− 6.7	− 6.9	− 7.8
1CVU	− 8.0	− 9.2	− 7.7	− 7.5
3V99	− 6.1	− 6.3	− 6.1	− 5.7

**Figure 5:**
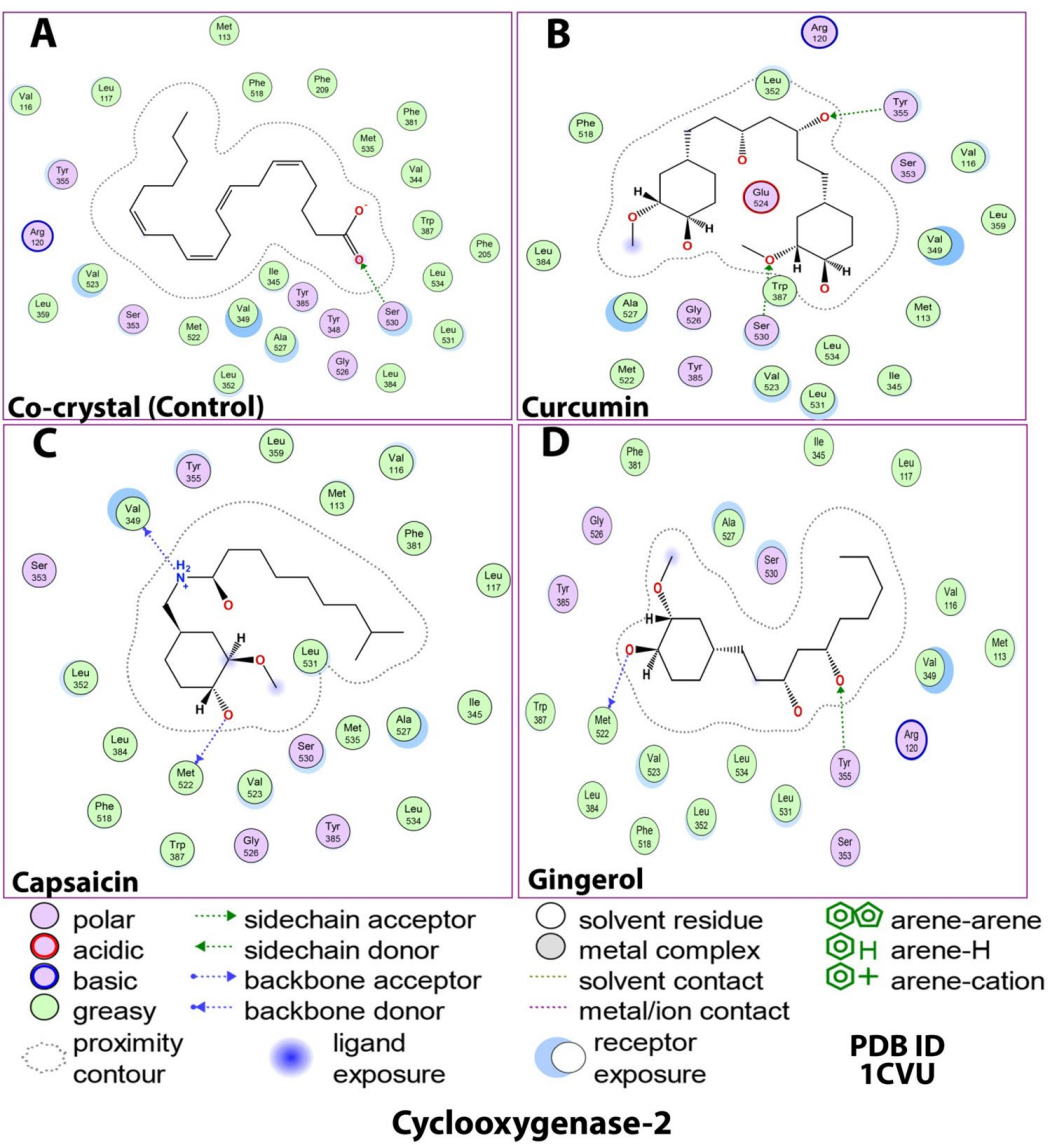
2D docking interaction of ligands: (**A**) Internal/co-crystallized ligand, (**B**) Curcumin, (**C**) Capsaicin, and (**D**) Gingerol in the binding pocket of COX-2 (PDB ID: 1CVU) showing various interactions.

**Figure 6:**
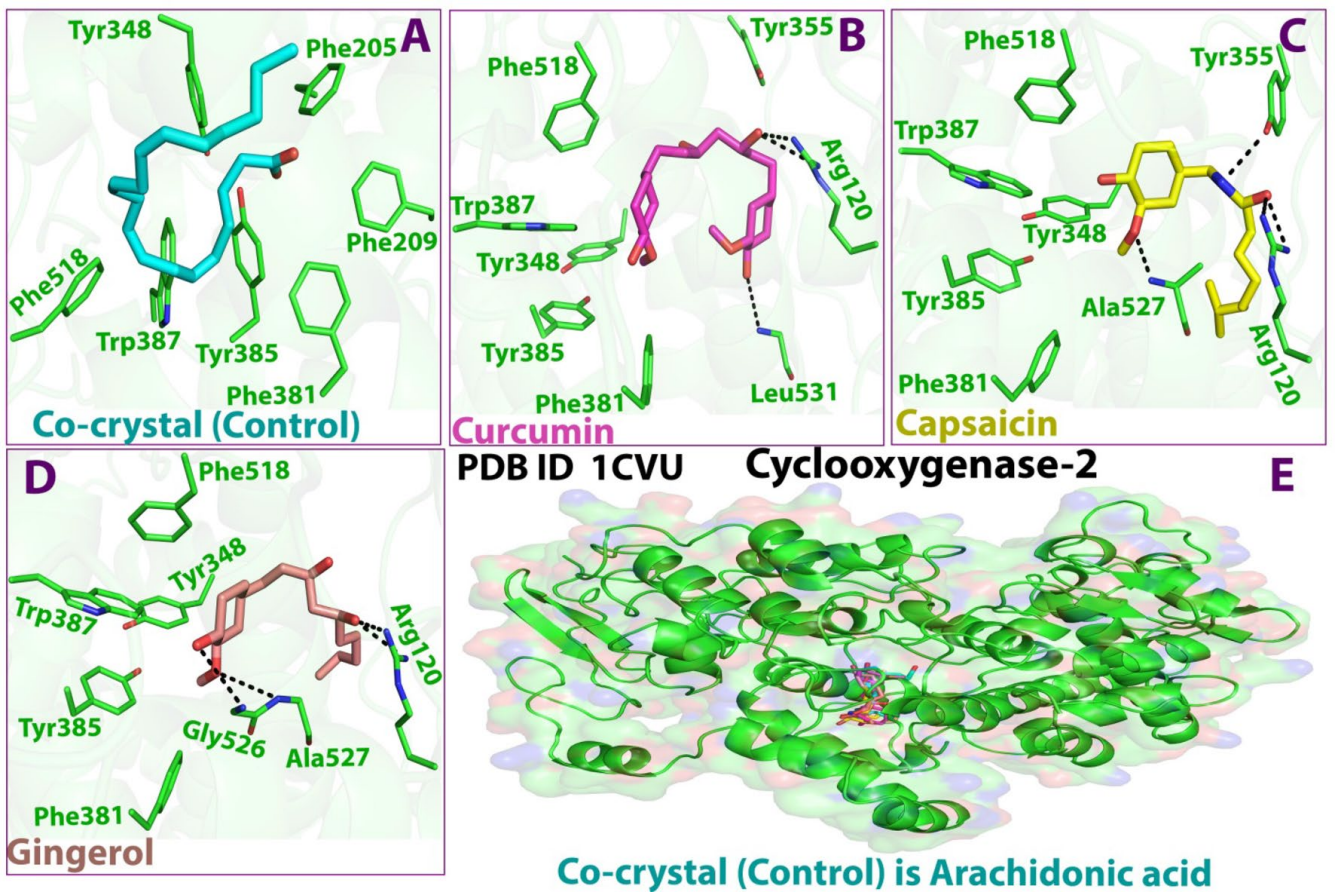
3D docking interaction of ligands: (**A**) Internal/co-crystallized ligand, (**B**) Curcumin, (**C**) Capsaicin, and (**D**) Gingerol in the binding pocket of COX-2 (PDB ID: 1CVU) showing various interactions. (**E**) Ribbon representation of cyclooxygenase-2 Protein.

Structurally, there were relative correlations among the number of H-bonds. Upon binding with 1CVU, curcumin had two H-bonds with Ser530 and Tyr355, gingerol had two H-bonds with Met522 and Tyr335, and capsaicin had two H-bonds with Val349 and Met522 (Figs. 5 and 6; Table 4). In this case, the co-crystallized ligand made only one H-bond with Ser530.

**Table 4:** Details of docking interaction of curcumin, capsaicin, and gingerol with the interacting residues in the binding pocket of COX-2 (PDB ID: 1CVU).

Ligand	(PDB ID: 1CVU) docking interactions			
	H-bond & type	Distance (Å)	Energy (kcal/mol)	Other interacting residues
Co-crystallized	Ser530 H-acceptor	2.75	− 3.4	Hydrophobic greasy: Met113, Phe516, Phe209, Met535, Phe381, Val344, Trp387, Phe205, Leu534, Leu531, Leu384, Ile345, Leu352, Met522, Leu359, Leu117 Polar: Tyr355, Arg120, Ser353, Tyr385, Tyr348, Gly526
Curcumin	Ser530, Tyr355 H-acceptor	2.77 & 2.81	− 1.8, − 1.6	Hydrophobic greasy: Val116, Leu359, Val349, Met113, Ile345, Leu534, Leu531, Val523, Trp387, Met522, Ala527, Leu384, Phe518 Polar: Arg120, Ser353, Tyr385, Gly526, Glu524
Capsaicin	Val349, Met522 H-donor	3.10 & 2.80	− 5.2, − 3.1	Hydrophobic greasy: Leu359, Met113, Val116, Phe381, Leu117, Ile345, Leu534, Ala527, Met535, Val523, Trp387, Phe518, Leu384, Leu352 Polar: Tyr355, Ser530, Tyr385, Gly526, Ser353
Gingerol	Met522 & Tyr355 H-donor & acceptor	2.80 & 2.90	− 3.0 & − 2.0	Hydrophobic greasy: Phe381, Ala527, Ile345, Leu117, Val116, Val349, Met113, Leu531, Leu534, Leu352, Val523, Phe518, Leu384, Trp387 Polar: Gly526, Tyr385, Ser530, Arg120, Ser353

Due to the high homology of binding site residues, various interactions were conserved. The high docking score of curcumin could be attributed to the formation of H-bonds and different other interactions, i.e., hydrophobic and polar. Apart from conserving the H-bond with Ser530, curcumin was able to make a new H-bond with Tyr355. This new H-bond formation might allow deeper penetration into COX-2 protein. It has also been observed that the COX-2 binding site was 20% larger than that of COX-1. Apart from establishing H-bonds, it also showed varied hydrophobic interactions with a variety of amino acid residues (Val116, Leu359, Val349, Met113, Ile345, Leu534, Leu531, Val523, Trp387, Met522, Ala527, Leu384, Phe518). These interactions play a major role in stabilizing the ligands at the binding interface. Moreover, the strong binding affinity could be attributed to the presence of polar residues, namely Arg120, Ser353, Tyr385, Gly526, and Glu524 that enhance the chances of squeezing the molecule into the active site. To sum up, curcumin’s selectivity to COX-2 was primarily due to the binding to the hydrophobic region and extending to the lobby region near the entrance of the COX-2 binding site, thereby forming hydrogen bonds with Ser530^69^. Moreover, the position of the co-crystallized ligand and the three test compounds inside the catalytic site of COX-2 was stabilized by surrounded hydrophobic interactions.

**Figure 7:**
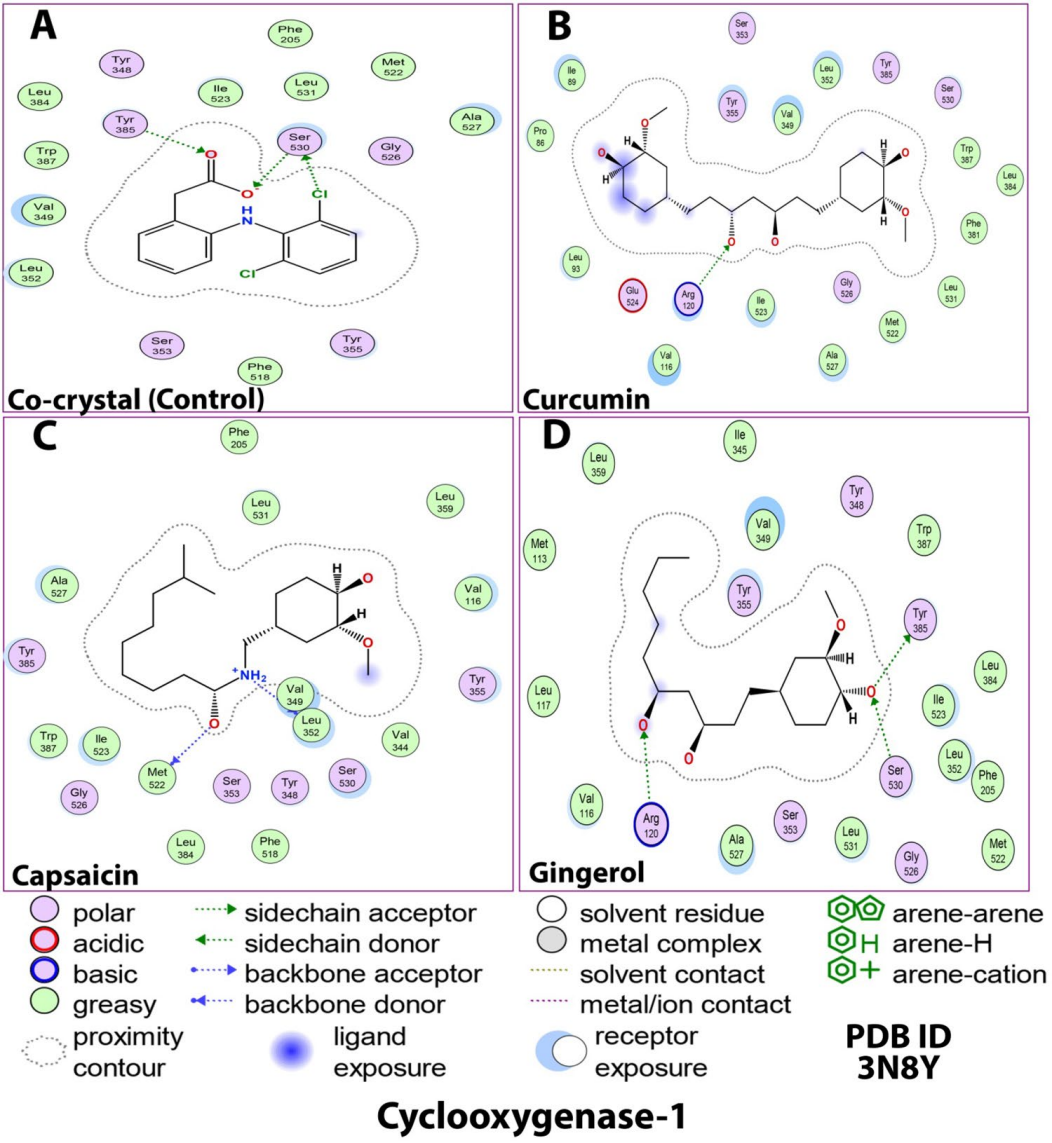
2D docking interaction of ligands: (**A**) Internal/co-crystallized ligand, (**B**) Curcumin, (**C**) Capsaicin, and (**D**) Gingerol in the binding pocket of COX-1 (PDB ID: 3N8Y) showing various interactions.

**Figure 8:**
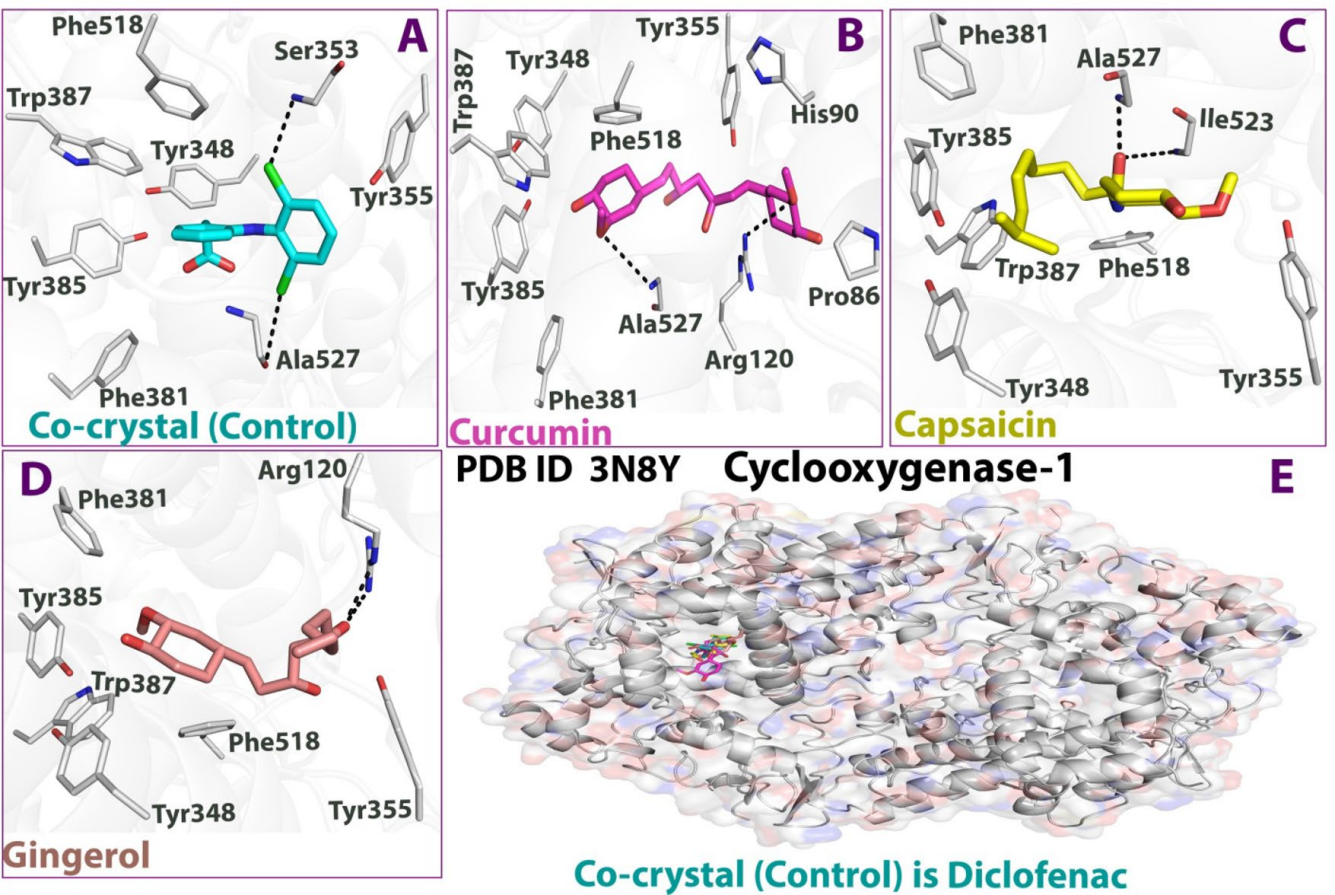
3D docking interaction of ligands: (**A**) Internal/co-crystallized ligand, (**B**) Curcumin, (**C**) Capsaicin, and (**D**) Gingerol in the binding pocket of COX-1 (PDB ID: 3N8Y) showing various interactions. (**E**) Ribbon representation of cyclooxygenase-1protein.

To understand the binding modes of ligands in COX-1, it is crucial to have a detailed analysis of the various interactions at the residues level. Figures 7 and 8; Table 5 highlights all the significant interactions made by the ligands in the active site of the proteins. In the open conformational environment of protein structures, energetically favored ligands are stabilized through weak intermolecular interactions such as hydrogen bonding and hydrophobic interactions. The bound internal ligand showed two H-bonds with Ser530 and Tyr385. These H-bonds were conserved very well in all the ligands under study. The binding of curcumin with 3N8Y revealed one H-bond with Arg120, whereas capsaicin and gingerol made two (Leu352 and Met522) and three (Tyr385, Ser530 and Arg120) H-bonds, respectively. As per the docking score presented in Table 4, curcumin showed a higher negative score than the internal ligand signifying a relatively better COX-1 inhibition. Moreover, the H-bond with Arg120 plays a significant role, as suggested by mutational analyses that an interaction of the carboxylate of arachidonic acid with Arg-120 is required for high-affinity binding by COX-1 in its catalytically productive conformation (hydrophobic groove above Ser530) as arachidonic acid is a preferred substrate for COX-1^70^.

**Table 5:** Details of docking interaction of curcumin, capsaicin, and gingerol with the interacting residues in the binding pocket of COX-1 (PDB ID: 3N8Y).

Ligand	(PDB ID: 3N8Y) docking interactions			
	H-Bond & type	Distance (Å)	Energy (kcal/mol)	Other interacting residues
Co-crystallized	Ser530, Tyr385 H-donor, H-acceptor & H-acceptor	3.36, 2.53, & 2.68	− 0.7, − 1.0, − 1.5	Hydrophobic greasy: Phe205, Ile523, Leu531, Met522, Ala527, Phe518, Leu352, Val349, Trp387, Leu384 Polar: Tyr348, Gly526, Tyr355, Ser353
Curcumin	Arg120 H-acceptor	2.91 & 3.18	− 3.8, − 1.4	Hydrophobic greasy: Val349, Leu352, Trp387, Leu384, Phe381, Leu531, Met522, Ala527, Ile523, Val116, Leu93, Pro86, Ile89 Polar: Ser353, Tyr355, Tyr385, Ser530, Gly526, Glu524
Capsaicin	Leu352, Met522 H-donor	2.81 & 2.59	− 5.4, − 2.7	Hydrophobic greasy: Phe205, Leu531, Leu359, Val116, Val344, Val349, Phe518, Leu384, Ile523, Trp387, Ala527 Polar: Tyr385, Gly526, Ser353, Tyr348, Ser530, Tyr355
Gingerol	Tyr385, Ser530 & Arg120 H-donor, H-acceptor & H-acceptor	2.86, 2.78, & 2.96	− 0.9, -1.8, − 3.5	Hydrophobic greasy: Ile345, Val349, Trp387, Ile523, Leu384, Leu352, Phe205, Met522, Leu531, Ala527, Val116, Leu117, Met113, Leu359 Polar: Tyr355, Tyr348, Gly526, Ser353

The curcumin ligand’s backbone makes various hydrophobic contacts, viz. Val349, Leu352, Trp387, Leu384, Phe381, Leu531, Met522, Ala527, Ile523, Val116, Leu93, Pro86, and Ile89, and few polar interactions viz. Ser353, Tyr355, Tyr385, Ser530, Gly526, and Glu524. This encloses the curcumin molecule in a cage-like structure, justifying its COX-1 inhibition and increasing its selectivity^69^. Capsaicin and gingerol displayed less binding energies and interactions with the COX-1 binding site. This could be attributed to the incompatibility between the narrow groove (Arg120 to Tyr355) and the bulkiness of the molecules. There might be an inconsistency in their positions and interaction patterns concerning curcumin and internal ligand. Based on the analysis of interactions, curcumin seems to inhibit COX-1 prominently.

**Figure 9:**
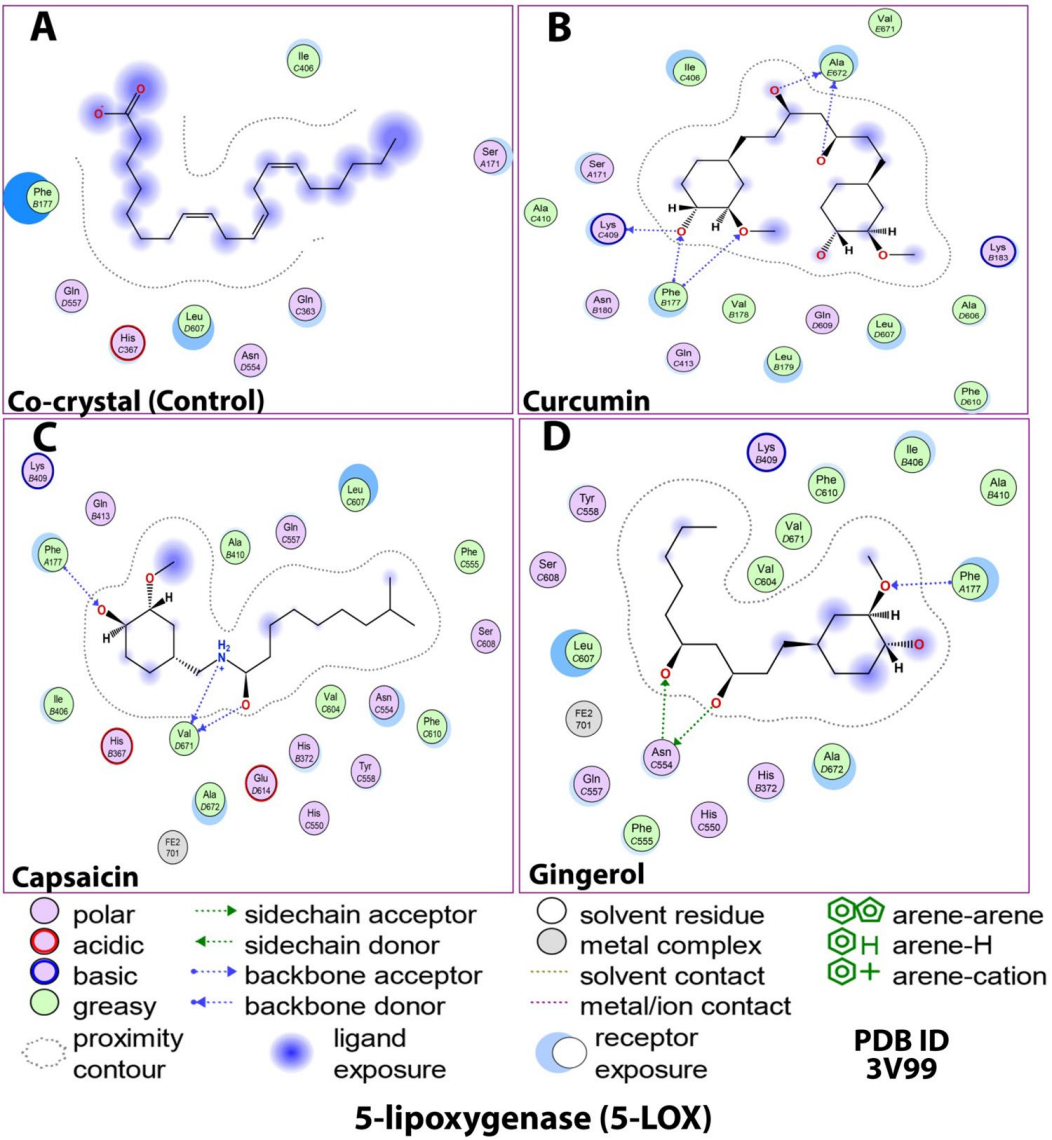
2D docking interaction of ligands: (**A**) Internal/co-crystallized ligand, (**B**) Curcumin, (**C**) Capsaicin, and (**D**) Gingerol in the binding pocket of 5-LOX (PDB ID: 3V99) showing various interactions.

**Figure 10:**
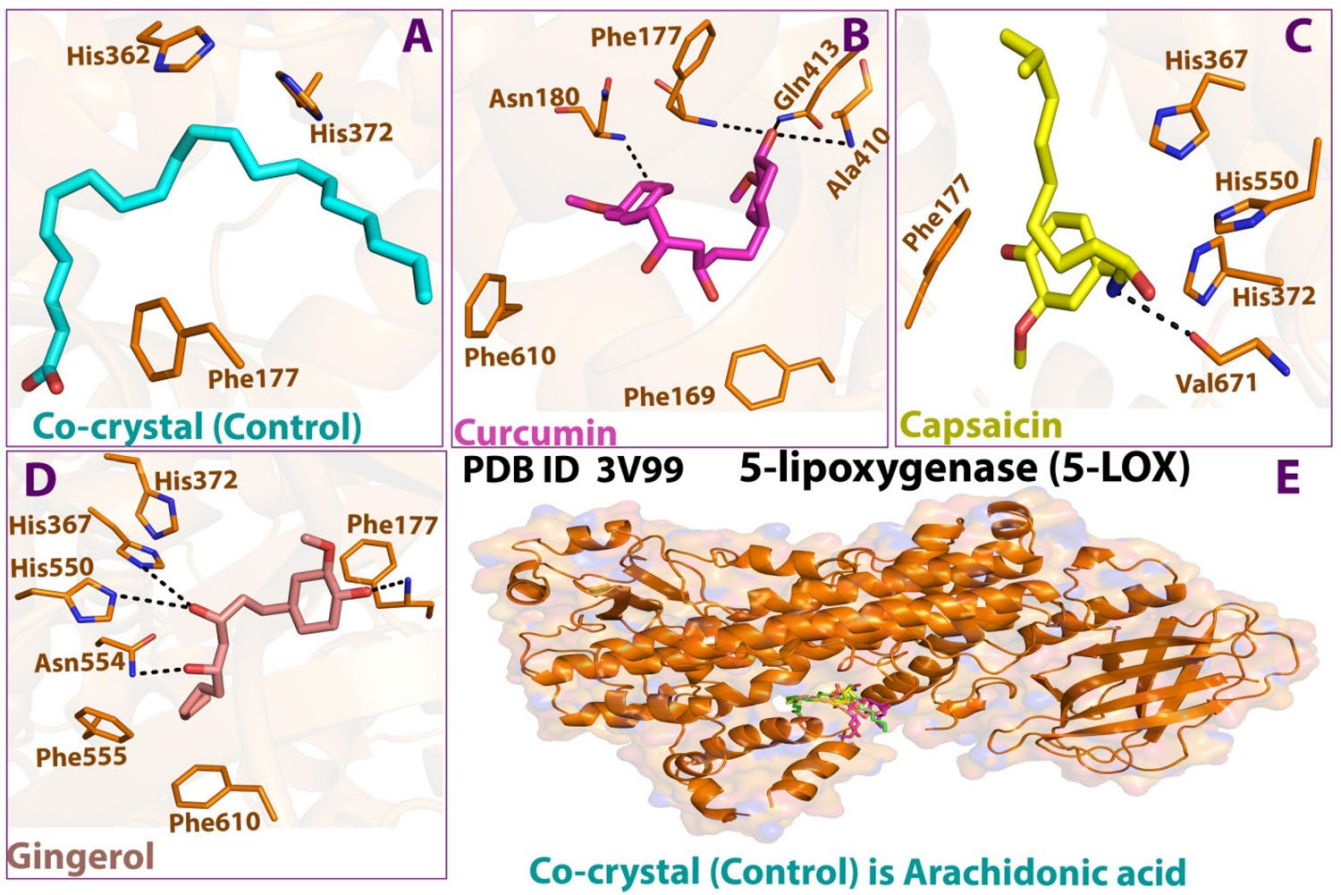
3D docking interaction of ligands: (**A**) Internal/co-crystallized ligand, (**B**) Curcumin, (**C**) Capsaicin, and (**D**) Gingerol in the binding pocket of 5-LOX (PDB ID: 3V99) showing various interactions. (**E**) Ribbon representation of cyclooxygenase-2 Protein.

The 5-lipoxygenase (5-LOX) enzyme is responsible for the initiation of biosynthesis of pro-inflammatory leukotriene lipid mediators and the synthesis of anti-inflammatory lipoxins^71^. Figures 9 and 10; Table 6 highlights the interactions made by the ligands with the amino acid residues of the 5-LOX protein. Looking into the structure of 5-LOX, the residues 1–112 form the primarily beta-sheeted N-terminal regulatory domain, and the residues 125–673 constitute the much larger C-terminal catalytic part. The catalytic site of this domain is nested around a catalytic iron (Fe^+2^) which forms the center of the active site. The active site cavity appears to have a partial U-shaped cavity lined with highly conserved Leu and Ile residues. However, 5-LOX protein has a unique catalytic cavity with no substrate access site due to the shielding effects of outer solvents. It comprises the side chains of residues Phe177 and Tyr181 positioned to impede access into the catalytic cavity. The presence of catalytic iron in 5-LOX acts as both an electron donor andacceptor^72^.

**Table 6:** Details of docking interaction of curcumin, capsaicin, and gingerol with the interacting residues in the binding pocket of 5-LOX (PDB ID: 3V99).

Ligand	(PDB ID: 3V99) Docking Interactions			
	H-Bond& type	Distance (Å)	Energy (kcal/mol)	Other interacting residues
Co-crystallized	–	–	–	Hydrophobic greasy: Ile406, Leu607, Phe177 Polar: Gln557, His367, Asn554, Gln363, Ser171
Curcumin	Lys409, Ala672, Phe177 H-donor, H-acceptor	2.71, (2.76, 2.96), (3.21, 2.80)	− 1.5, (− 1.8, − 1.5), (− 1.5, − 3.0)	Hydrophobic greasy: Ile406, Val671, Ala606, Phe610, Leu607, Leu179, Val178, Ala410 Polar: Lys183, Gln609, Gln413, Asn180, Ser171
Capsaicin	Val671, Phe177 H-donor, H-acceptor	(2.87, 2.77), 2.88	(− 11.1, − 1.3), − 7.3	Hydrophobic greasy: Ala410, Leu607, Phe555, Phe610, Val604, Ala672, Ile406 Polar: Lys409, Gln413, Gln557, Ser608, Asn554, His372, Tyr558, His550, Glu614, His367 Metal complex: Fe^+2^ 701
Gingerol	Asn554 & Phe177 H-donor, H-acceptor & H-acceptor	(2.65, 2.83) & 2.80	(− 1.7, − 2.7), − 8.7	Hydrophobic greasy: Val604, Val671, Phe610, Ile406, Ala410, Ala672, Phe555, Leu607 Polar: Lys409, His372, His550, Gln557, Ser608, Tyr558 Metal complex: Fe^+2^ 701

Compared to the internal ligand, wherein hydrogen bonds were formed, curcumin, capsaicin, and gingerol were interactive enough to establish H-bonds. Curcumin had three H-bonds with Lys409, Ala672, and Phe177, capsaicin had two H-bonds with Val671 and Phe177; and gingerol had two H-bonds with Asn554 and Phe177. These interactions help the ligands access the active site via the α2 helix. The H-bond with Phe177 is critical in providing a fully functional active site.Fe^+2^ cofactor of the 5-LOX enzyme was bound to capsaicin and gingerol, which causes the chelation of these molecules. Besides, all ligands were mostly enclosed around hydrophobic residues. These hydrophobic interactions (Ile406, Leu607, Ala410, and Phe610) were primarily conserved in all cases, along with polar interactions (histidine and serine residues). Though all ligands bind effectively with 5-LOX, the number of H-bonds made by curcumin is more than other ligands under study. It was stabilized by surrounded hydrophobic and polar interactions, contributing to a slightly higher 5-LOX inhibition than different ligands. Hence, curcumin effectively binds to the active site and inhibits 5-LOX activity that might reduce leukotriene production thereby reducing the inflammatory responses.

To conclude, among all three ligands under study, curcumin could be considered the best-fit molecule for all three proteins. The geometry of curcumin allows its insertion into the active site of all three proteins, with hydrogen bonding potential, i.e. acceptor/donor groups that improve the affinity for COX-1/2 and 5-LOX. From the above discussion, it is evident that curcumin has the potential to block both COX-1/2 and the 5-LOX enzymes. It generally acts by blocking the formation of both prostaglandins and leukotrienes, but does not affect lipoxin formation. Such combined inhibition might avoid some disadvantages of selective COX-2 inhibitors and spares the gastrointestinal mucosa.

### Predictive AMDET analysis

#### ADME

All three ligands under study were evaluated for their ADMET profile using online web server i.e. Swiss ADME (http://www.swissadme.ch/). The obtained ADME properties are presented in detail in Table 7. The destiny of a compound in the human body is often evaluated in terms of ADME properties, as it highlights the behavior of the molecule in the human body^73–75^. Results indicated that the molar refractivity, which accounts for the overall polarity of the molecules, was 102.80, 90.52, and 84.55 for curcumin, capsaicin, and gingerol, respectively; these values were in the normal range (30–140)^76^.The topological polar surface area (TPSA) was 93.06, 58.56 and 66.76 Å^2^ for curcumin, capsaicin, and gingerol, respectively. This refers to the molecule’s ability to permeate cells, i.e. molecules with TPSA > 140 Å^2^ are poor at cell permeation. A TPSA < 90 Å^2^ is usually preferred for molecules to permeate the BBB and act on CNS^76^. This suggests that curcumin cannot cross the blood–brain barrier (BBB), while the other two can.Regarding drug properties that affect ADMET, “solubility class lipophilicity” refers to a molecule's ability to dissolve into a lipophilic medium^77^. These properties include permeability, ADME, solubility, plasma protein binding, and toxicity. Results of iLOGP^78^ and SILICOS-IT(an hybrid method relying on 27 fragments and 7 topological descriptors (http://silicos-it.be.s3-website-eu-west-1.amazonaws.com/software/filter-it/1.0.2/filter-it.html, accessed through Swiss ADME website on November 2022) suggested that the iLOGP values of all the molecules were in the acceptable range (− 0.5 to + 5.4), while SILICOS-IT values for all the identified leads were in the most favorable range (− 10 to 0). Water solubility is a significant factor determining a drug’s distribution and absorption. Log S calculations show the molecule’s solubility in water at 25 °C. The ESOL model’s calculated log S values shouldn’t be more significant than six for appropriate solubility^79^. Curcumin, capsaicin, and gingerol showed log S values of − 3.94, − 3.53, and − 2.96, accounting for a good solubility profile. According to the findings above, all of the lead compounds showed a good balance between permeability and solubility and may exhibit acceptable bioavailability when taken orally. All the molecules revealed a high predicted GI absorption^80^. Understanding the results of ADMET and cell-based bioassays is aided by permeability predictions^81^. Results indicated that the permeability over human skin was -6.28, -5.62, and -6.14 cm/s for curcumin, capsaicin, and gingerol, respectively. These predicted values were in the acceptable range^81^.Drug interactions caused by metabolism can occasionally reduce a drug’s bioavailability. Drug-metabolizing enzymes can only bind to the drug in its free form. As the most significant class of metabolizing enzymes, cytochrome P450 enzymes (CYPs) must be studied to understand the metabolic behavior of studied molecules. The CYPs (CYPs of human liver microsomes (HLM)) inhibitory activity of all the lead compounds was evaluated^82^. Only curcumin showed a good profile in this regard. A molecule's drug-likeness indicates whether it has the potential to develop into an oral medication.

**Table 7:** Description of in silico ADME parameters of all three ligands under study.

	Compounds code		Curcumin	Capsaicin	Gingerol
ADME profile	Physiochemical parameters	Formula	C_21_H_20_O_6_	C_18_H_27_NO_3_	C_17_H_26_O_4_
		Molecular weight	368.38 g/mol	305.41 g/mol	294.39 g/mol
		Mol. refractivity	102.80	90.52	84.55
		TPSA	93.06 Å^2^	58.56 Å^2^	66.76 Å^2^
	Lipophilicity	ILOGP	3.27	3.15	3.48
		SILICOS-IT	− 4.04	− 4.10	− 4.04
	Water solubility	Log S (ESOL), class	− 3.94 Soluble	− 3.53 Soluble	− 2.96 Soluble
	Pharmacokinetics	GI absorption	High	High	High
		Plasma protein binding (human)	70.35	79.22	94.56
		BBB permeant	No	Yes	Yes
		Log K_p_(skin perm.)	− 6.28 cm/s	− 5.62 cm/s	− 6.14 cm/s
		CYP1A2	No	Yes	Yes
		CYP2D6	No	Yes	Yes
	Drug-likeness rules	Lipinski (Pfizer)	Yes	Yes	Yes
		Ghose (Amgen)	Yes	Yes	Yes
		Veber (GSK)	Yes	Yes	Yes
		Egan (Pharmacia)	Yes	Yes	Yes
		Muege (Bayer)	Yes	Yes	Yes
		Bioavailability score	0.55	0.55	0.55
	Medicinal Chemistry	PAINS	0 alert	0 alert	0 alert
		Brenk	2 alerts	1 alert	2 alerts
		Synthetic accessibility	2.97	2.32	2.97

**Figure 11:**
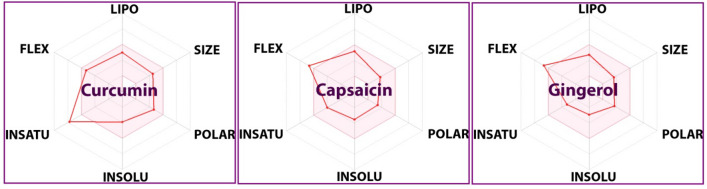
Illustration of computed bioavailability chart of curcumin, capsaicin, and gingerol.

In our investigation, the drug-likeness was determined using five distinct filters. All molecules displayed no violation of drug-likeness rules and had a bioavailability score of 55% (good bioavailability). The Abbot Bioavailability score, which can be determined by a feasibility score of 11%, 17%, 56%, or 85%, forecasts the fate of a chemical for an experiment including quantifiable Caco-2 cell line permeability or 10% oral bioavailability (in rats)^83^.The computed bioavailability chart of the phytocompounds under study has been presented in Fig. 11 in a spider web-like image. To identify potential ambiguous regions that result in false-positive biological output, PAINS, and Brenk techniques were used^84,85^. All molecules did not violate PAINS, while few violations were seen in Brenk. For all molecules, the synthetic accessibility was also estimated^86^. According to the criteria, all compounds displayed a moderate level of hardness (on a scale of 1 (easy) to 10 (very tough)).Thus, from the above data, it is evident that all molecules predicted ADME data are within the recommended values, with curcumin being the best.

#### Toxicity

All three molecules (curcumin, capsaicin, and gingerol) under study were evaluated for their toxicity profile using an online web server, pkCSM (https://biosig.lab.uq.edu.au/pkcsm/prediction). The AMES test, which uses microorganisms to determine a chemical compound's projected mutagenic potential, produced positive results for all molecules, revealing no AMES toxicity. The most significant dose that most patients can accept is technically known as the maximally tolerated dose (MTD). Herein the maximum tolerated dose (human) was 0.081, 0.46, and 0.0635 Log mg/kg/day for curcumin, capsaicin, and gingerol, representing a moderate dosage level per protocols, with curcumin being the most potent one. hERGI and II (human Ether-a-go-go-Related gene) codes for proteins that regulate ion channels responsible for the cardiac electrical action potential of the heart. Hence, drugs under study should prohibit their inhibition during drug development. Only curcumin showed no hERGI and II inhibition, negating ventricular arrhythmias' probability. For curcumin, capsaicin, and gingerol, the Oral Rat Chronic Toxicity (LD_50_) values were 1.833, 2.065, and 1.958 mol/kg, respectively, while the Oral Rat Chronic Toxicity (LOAEL) values were 2.228, 1.827, and 1.631, respectively, indicating a good safety profile. All molecules (curcumin and gingerol) except for capsaicin were predicted to be non-hepatotoxic. All molecules showed no skin sensitization as well. *T. Pyriformis* toxicity and Minnow toxicity were at acceptable levels. Results of the predicted toxicity of all molecules under study are shown in Table 8.

**Table 8:** Predicted toxicities of compounds.

Model name	Units	Compound		
		Curcumin	Capsaicin	Gingerol
AMES toxicity	Yes/No	No	No	No
Max. tolerated dose (human)	Log mg/kg/day	0.081	0.46	0.635
hERG I inhibitor	Yes/No	No	No	No
hERG II inhibitor	Yes/No	No	Yes	No
Oral rat acute toxicity (LD_50_)	Mol/kg	1.833	2.065	1.958
Oral rat chronic toxicity (LOAEL)	Log mg/kg_bw/day	2.228	1.827	1.631
Hepatotoxicity	Yes/No	No	Yes	No
Skin sensitisation	Yes/No	No	No	No
*T. Pyriformis* toxicity	Log ug/L	0.494	2.074	1.417
Minnow toxicity	Log mM	− 0.081	0.44	0.966

### Molecular dynamics simulation

Based on the results of molecular docking and predictive ADMET, it could be concluded that curcumin was the only molecule among all three that had the potential to be a better lead candidate for the combined inhibition of COX-1/2 and 5-LOX. To validate the docked pose of the curcumin-protein complex, a 150 ns molecular dynamics was performed. This complex’s simulated data was compared with the crystallized protein’s simulated data to see the dynamic changes that occurred during the simulation in the presence of the new ligand (curcumin). MD studies are usually implemented to study the nature of macromolecules or analyze the physical movements of atoms and molecules. The obtained simulation findings are discussed below.

#### 100 ns simulation of co-crystallized proteins (3N8Y, 1CVU and 3V99) with their internal ligand

##### Root mean square deviation (RMSD) analysis

The RMSD values were substantial for all protein–ligand conformations. RMSD analysis indicates whether the simulation has equilibrated around a fixed value. In complex A, the protein RMSD was stable throughout the simulation with minor fluctuations between (25–30 ns, 50–55 ns, and 75–80 ns). The variation was in the range of 1.6–2.8 Å.

**Figure 12:**
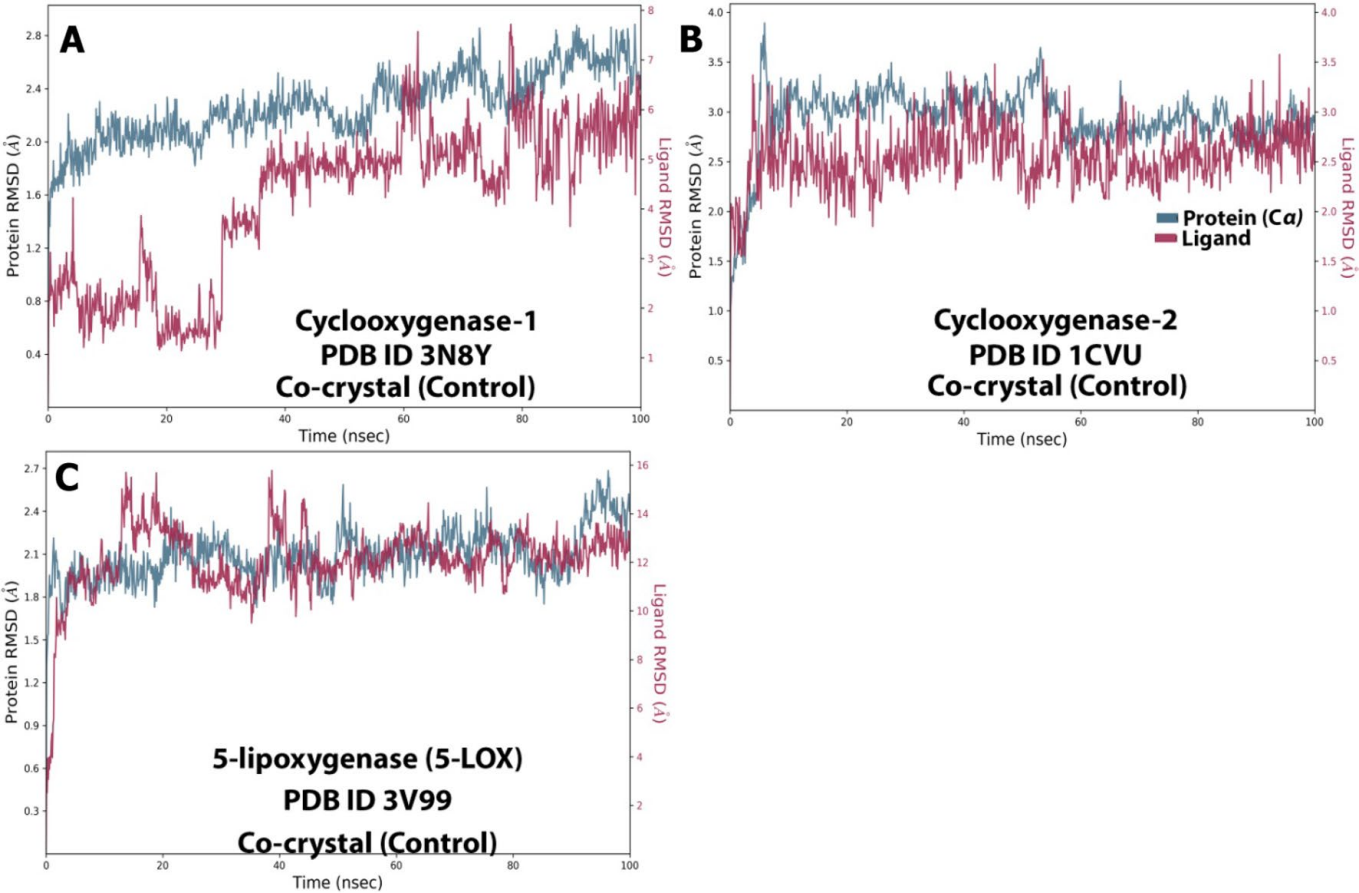
RMSD graph for 100 ns simulation trajectory of (**A**) 3N8Y/DIF complex, (**B**) 1CVU/ACD complex, and (**C**) 3V99/ACD complex.

How stable a ligand is in relation to a protein and its binding pocket is shown by the ligand RMSD (right Y-axis). Ligand RMSD was highly unstable as it showed significant fluctuations with 2.0–3.5 Å from 0 to 40 ns which then stabilized, showing 5–8 Å till 100 ns with some minor deviations (Fig. 12A). For complex B, there were the least variations in the trajectory. The Protein gained stability after 10 ns and showed a stable RMSD between 2.8 and 3.3 Å. Ligand gained stability after 5 ns and then was steady throughout the simulation with minor fluctuations in between, but RMSD variation was the least (2.5–2.8 Å) (Fig. 12B). The protein was highly stable for complex C as there were minor fluctuations in RMSD (1.8–2.2 Å) throughout the 100 ns simulation. The ligand was initially unstable but gained stability after 50 ns with an RMSD of 12–13 Å (Fig. 12C).

##### Root mean square fluctuation (RMSF) analysis

**Figure 13:**
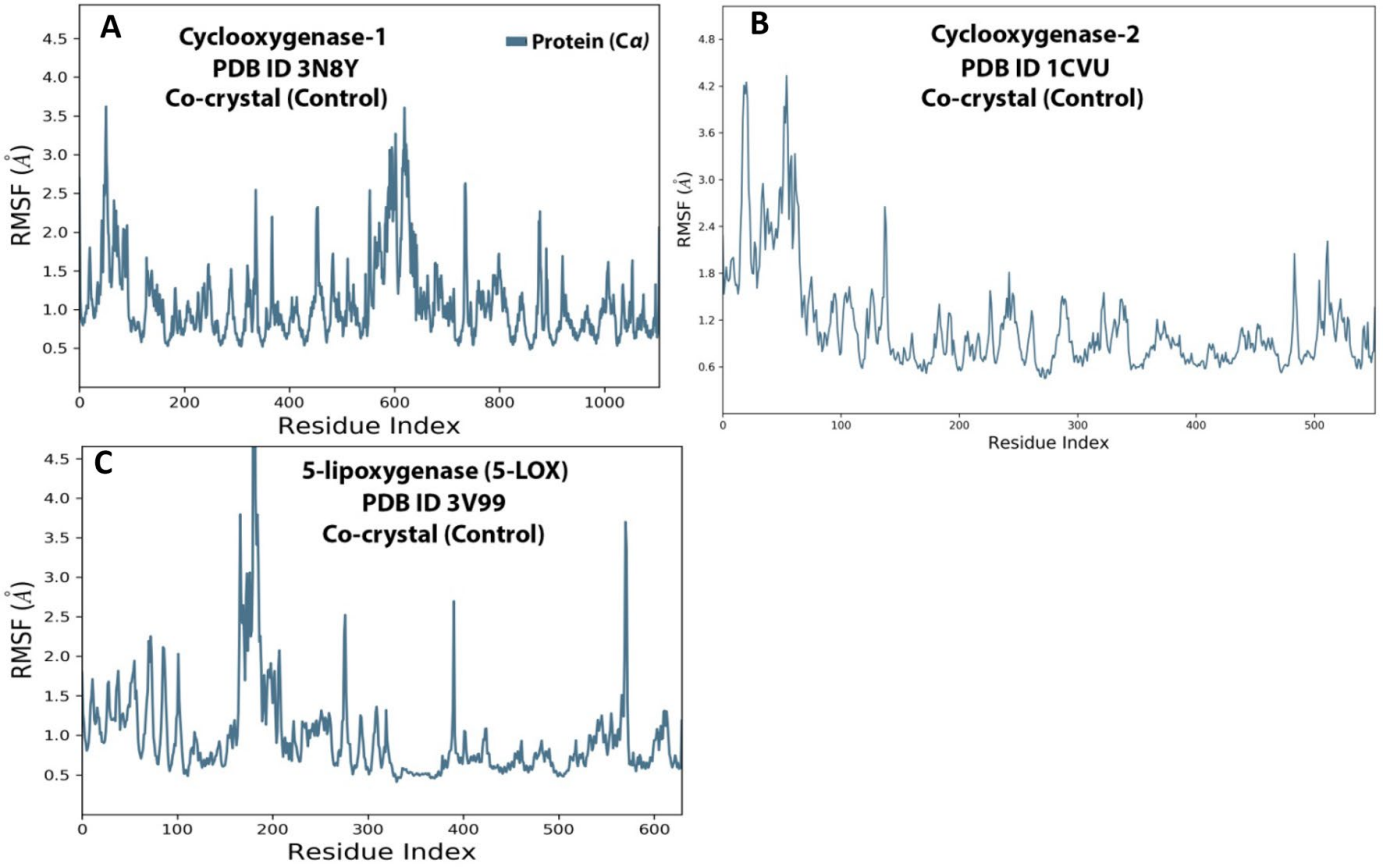
RMSF graphs for 100 ns simulation trajectory of co-crystal ligand-enzyme complexes. (**A**) 3N8Y, (**B**) 1CVU, and (**C**) 3V99.

For describing local variations along the protein chain, the RMSF is helpful. Peaks on this diagram represent regions of the proteins that vary most during the simulation. Usually, the protein’s N- and C-terminal tails move more than any other area. Because they are often more rigid than the protein's unstructured portion, parts of secondary structures like alpha helices and beta strands change less than loop sections. The RMSF of 3N8Y protein (Fig. 13A) looks stable, except amino acids from 3 to 100 and 580 to 620 showing higher fluctuations to3.5 Å, which are present in loops. For 1CVU protein (Fig. 13B), initially, there were fluctuations in amino acids from 1 to 100, but eventually, they gained stability. In the case of 3V99 protein (Fig. 13C), amino acid fluctuations were reduced from 180 to 200 (4.5 Å).

**Figure 14:**
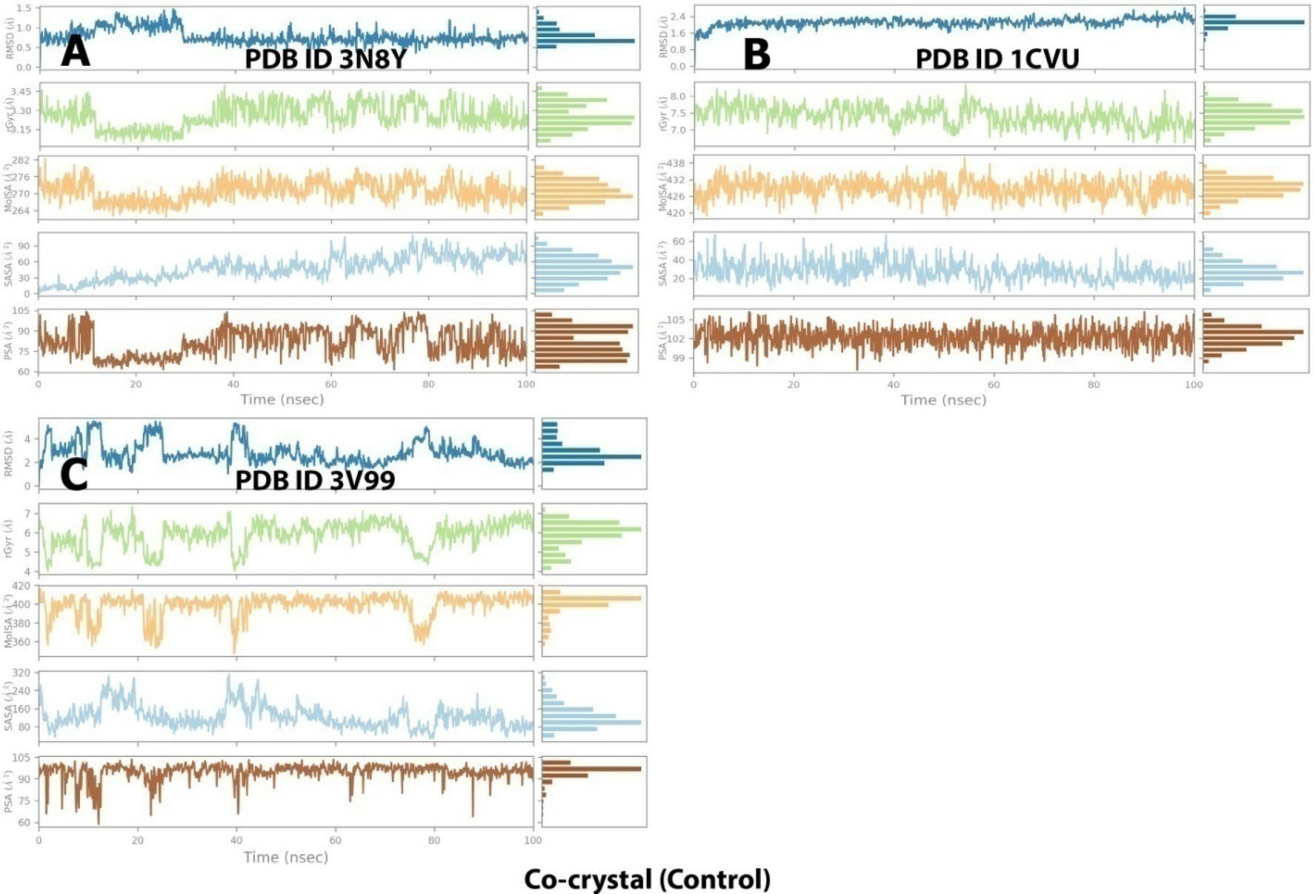
Trajectory of different properties of co-crystal ligand-enzyme complexes during the 100 ns of MD simulation. (**A**) 3N8Y, (**B**) 1CVU, and (**C**) 3V99.

The trajectories of different properties of co-crystal ligand-enzyme complexes (3N8Y, 1CVU, and 3V99) during the 100 ns of MD simulation are depicted in Fig. 14.

##### Protein–ligand contacts analysis

**Figure 15:**
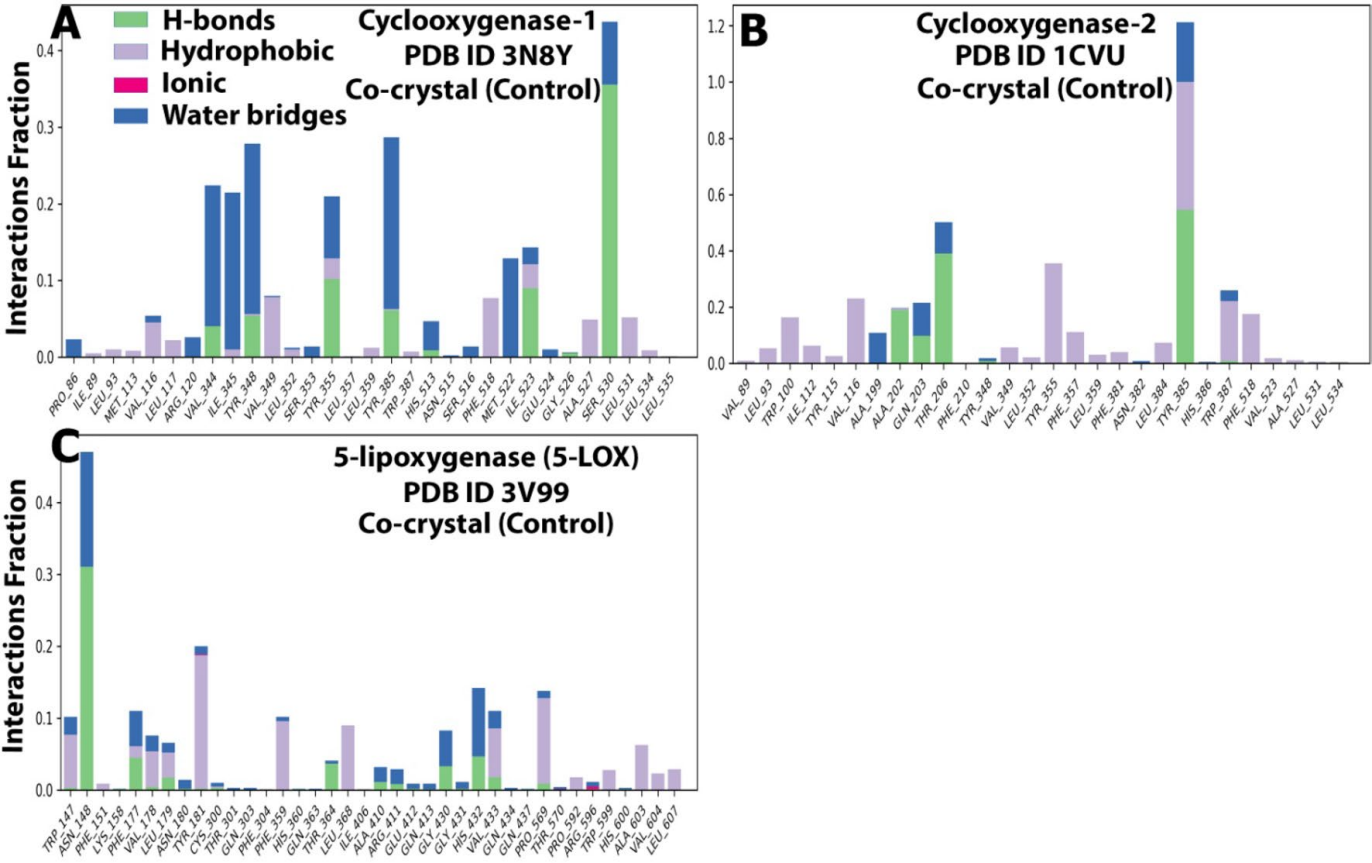
A plot of protein interactions with the ligand (stacked bar charts) was supervised throughout the molecular dynamics simulation of (**A**) 3N8Y/DIFcomplex, (**B**) 1CVU/ACD complex, and (**C**) 3V99/ACD complex.

Protein–ligand contacts were monitored throughout the simulation. H-bonds are essential for ligand binding. Because they have such a significant impact on drug specificity, metabolization, and adsorption. Hydrogen-bonding properties must be taken into account while developing new drugs. Herein complex A (Fig. 15A) showed H-bonding with Val344 (5%), Tyr348 (5%), Tyr355 (10%), Tyr385 (8%), Ile523 (10%), Ser530 (35%). Of these few H-bonds were conserved (Ser530) as seen in docking studies. Moreover, the internal ligand was positioned in the active pocket by forming hydrogen bonding, hydrophobic, and water-bridged interactions with Val344, Ile345, Tyr348, Met522, and Ala527. Complex B’s (Fig. 15B) position in the active site enabled it to form H-bonds with Ala202 (20%), Gln203 (10%), Thr206 (40%), and Tyr385 (50%). It also formed hydrophobic and water bridges with Trp100, Val116, Tyr355, Trp387, Phe518, and Phe357. Complex C (Fig. 15C) also positioned itself in the active site pocket and formed H-bonds with Asn148 (32%). It also formed hydrophobic and water bridges with Tyr181, Phe359, Leu368, Pro569, and Val433.

##### Interactions timeline

**Figure 16:**
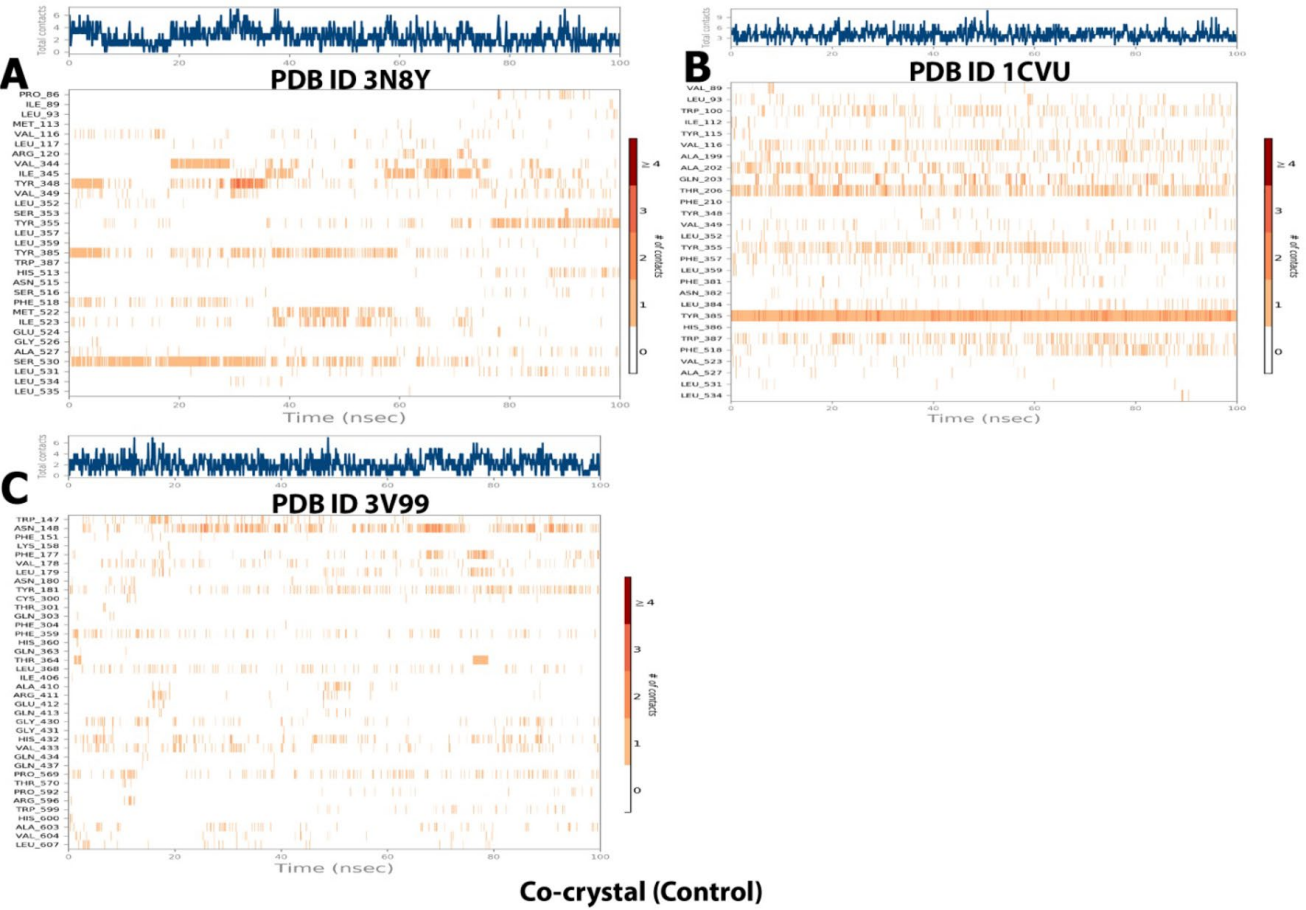
Specific contacts made by the proteins with the internal ligand throughout the simulation (dark color indicates more specific contact with the ligand) of(A)3N8Y/DIFcomplex, (B) 1CVU/ACD complex, and (C) 3V99/ACD complex.

The interactions and contacts (H-bonds, hydrophobic, ionic, and water bridges) presented andare shown in the form of a timeline in Fig. 16. The number of different interactions that the protein made with the ligand overall during the trajectory has been displayed in the top panel (dark blue). The residues that interact with the ligand in each trajectory frame are displayed in the bottom panel.According to the scale to the right of the plot, some residues have multiple specific contacts with the ligand, which is depicted by a darker orange color.

#### 150 ns simulation of proteins (3N8Y, 1CVU, and 3V99) with curcumin

##### RMSD analysis

**Figure 17:**
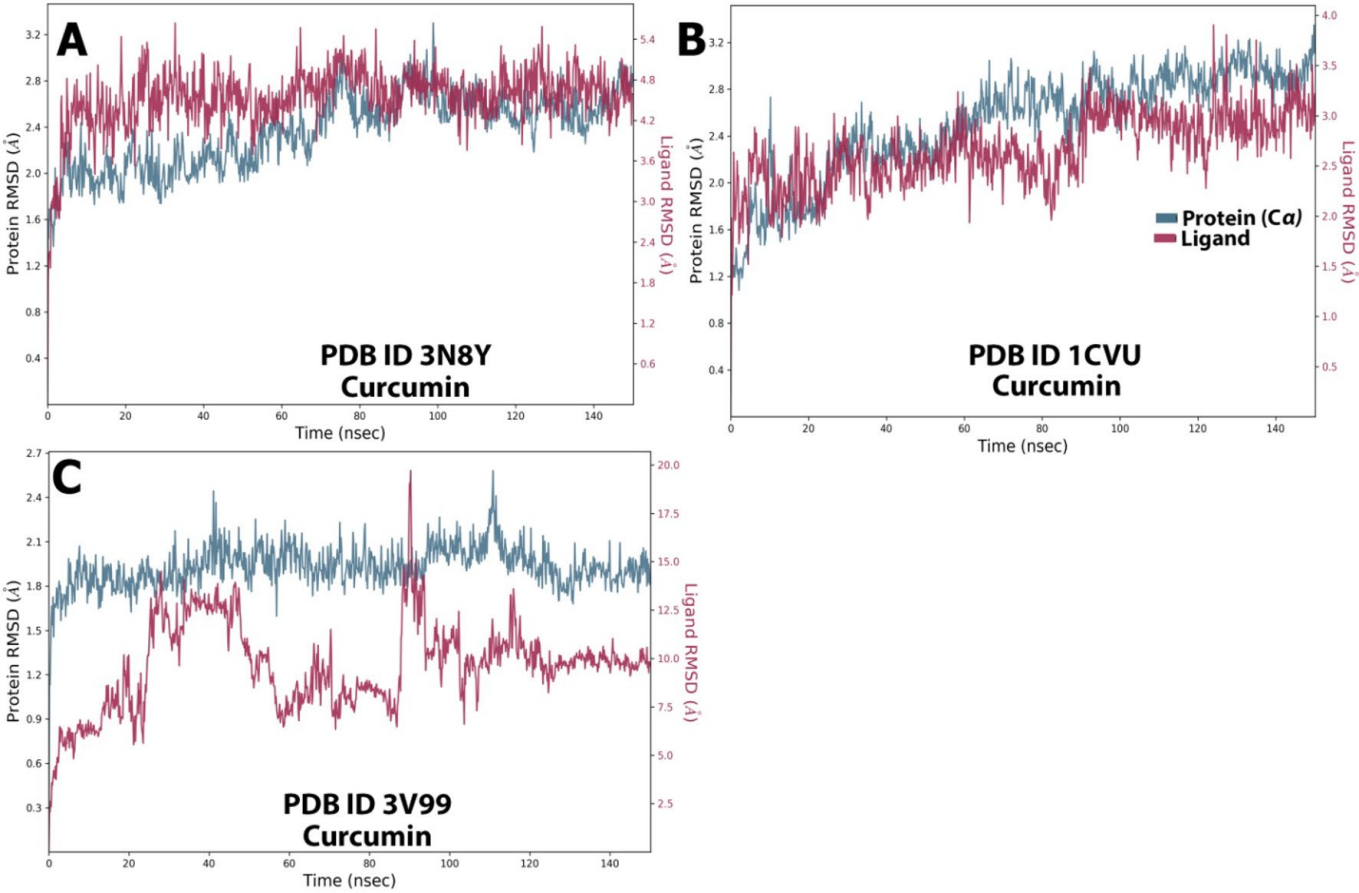
RMSD graph for 150 ns simulation trajectory of (**A**) 3N8Y/curcumin complex, (**B**) 1CVU/curcumin complex, and (**C**) 3V99/curcumin complex.

The average change in displacement of a particular set of atoms for a given frame relative to a reference frame is calculated using the RMSD values. For each frame in the trajectory, it is computed. The RMSD values are aligned with the protein-internal ligand confirmations. In complex A (Fig. 17A), the protein RMSD was stable throughout the simulation with minor fluctuations. Initially, the variation was in the range of 1.6–2.0 Å, but after 50 ns, a constant RMSD of 2.5 Å was achieved, which indicates protein's stability. In this case, curcumin’s RMSD (4.8 Å) was highly stable, with almost no fluctuations. This indicates the stability of the 3N8Y/curcumin complex. For complex B (Fig. 17B), initially, there were minor fluctuations in the protein (1.2–2.0 Å) upto 50 ns, after which it attained stability with RMSD of 2.8 Å till the end of the simulation. Hence this 1CVU/curcumin complex could also be considered stable. For complex C (Fig. 17C), the protein was highly stable (RMSD of 1.8–2.1 Å) with almost no deviations throughout the simulation, but the ligand (curcumin) displayed acceptable fluctuations. Fluctuations were observed between 30 and 50 ns (RMSD of 1.2–1.5 Å) and 85–105 ns (RMSD of 1.5–2.5 Å). Overall, the complex 3V99/curcumin can be considered stable.

##### RMSF analysis

RMSF analysis helps in understanding the local variations along the protein chain. Usually, peaks represent the residues that fluctuate the most during the simulation. The primary structures of a protein typically move more than the secondary structures as they are more rigid.

**Figure 18:**
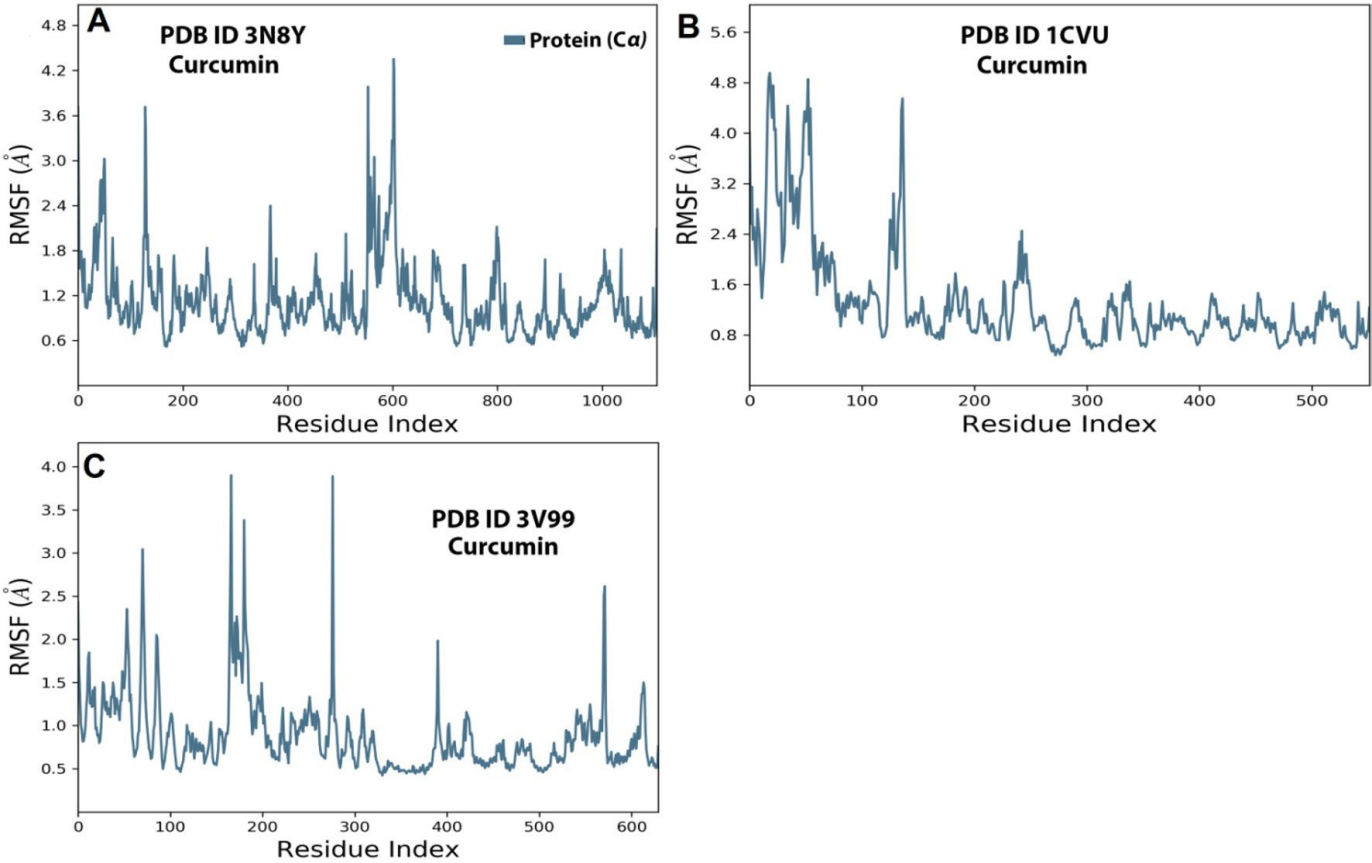
RMSF graph for 150 ns simulation trajectory of (**A**) 3N8Y, (**B**) 1CVU, and (**C**) 3V99.

The fluctuations in the protein-curcumin complex were almost the same as in the protein-internal ligand case. Hence this shows the complexes’ overall stability. Herein the RMSF of 3N8Y protein (Fig. 18A) looks stable except for amino acids from 150 to 160 and 580 to 620 showed higher fluctuations to3.5 Å. The overall residues showed an average RMSF value between 0.8 and 1.6 Å. For 1CVU Protein (Fig. 18B), there were fluctuations in amino acid residues (0–80 at 4.8 Å) during the early course of the simulation. Over time the protein gained stability and showed an RMSF value between 0.8 and 1.8 Å. In the case of 3V99 Protein (Fig. 18C), there were varied fluctuations in amino acids from 80 to 90 (3.0 Å), 180–200 (3.5 Å), and 575–590 (2.5 Å). To sum up, these minor fluctuations throughout the simulation are acceptable for small biomolecules.

**Figure 19:**
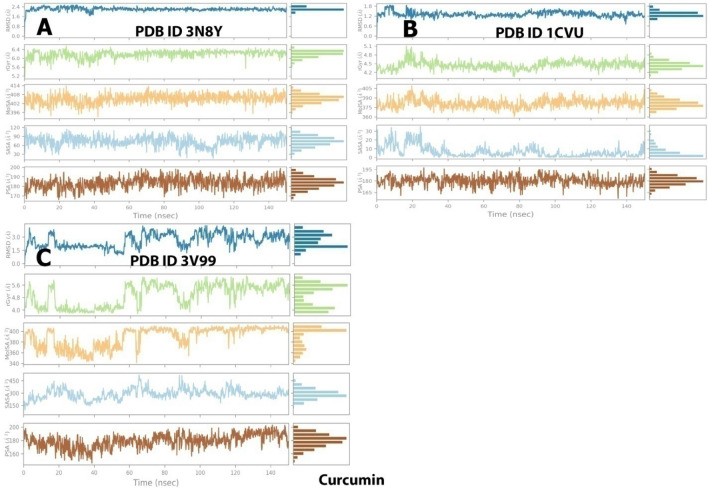
Trajectory of different properties of curcumin-enzyme complexes during the 150 ns of MD simulation. (**A**) 3N8Y, (**B**) 1CVU, and (**C**) 3V99.

The trajectories of different properties of curcumin-enzyme complexes (3N8Y, 1CVU, and 3V99) during the 150 ns of MD simulation are depicted in Fig. 19.

##### Protein–ligand contacts analysis

**Figure 20:**
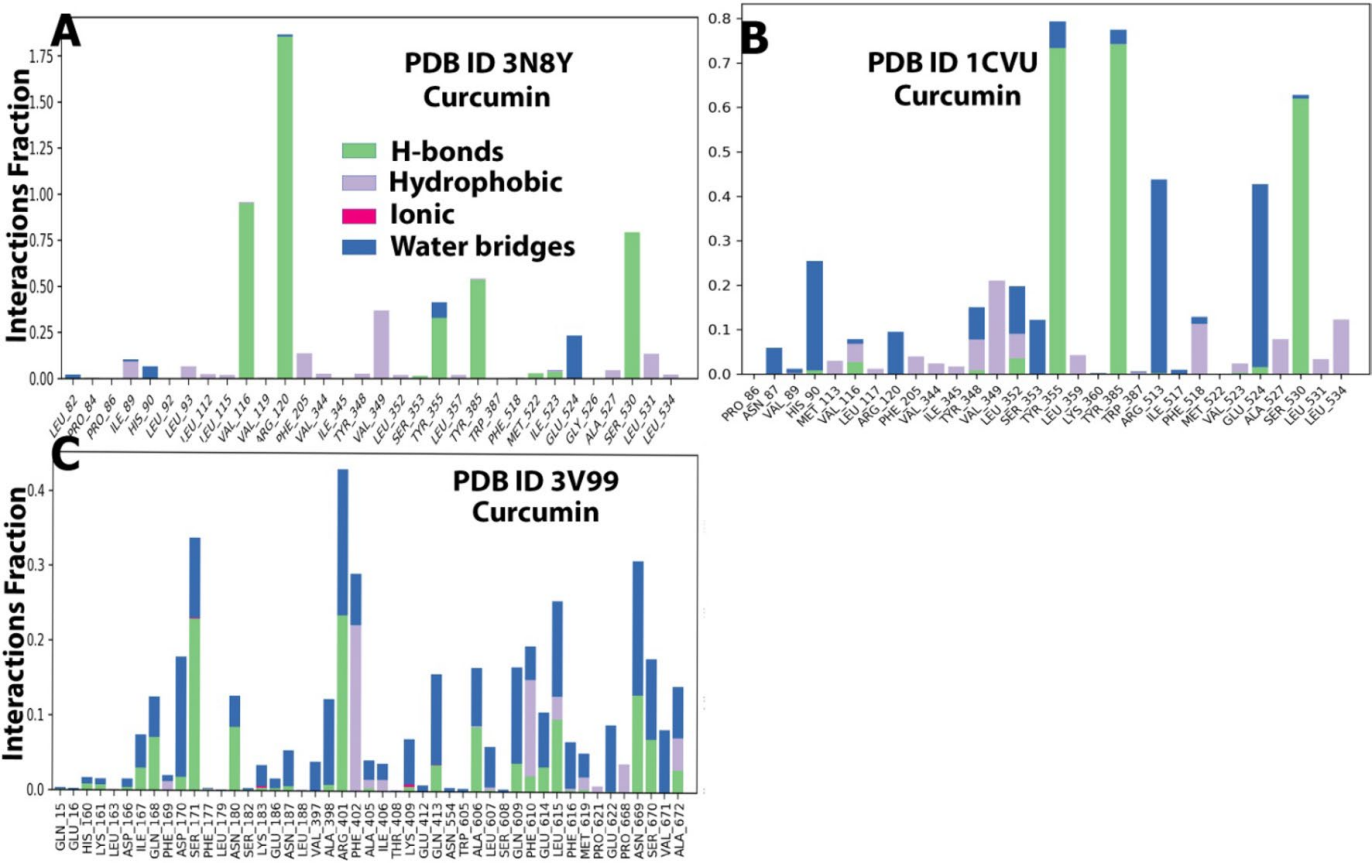
A plot of protein interactions with the ligand (stacked bar charts) supervised throughout the molecular dynamics simulation of the (**A**) 3N8Y/curcumincomplex, (**B**) 1CVU/curcumin complex, and (**C**) 3V99/curcumin complex.

Protein–ligand contacts refer to the interactions between the protein and ligand during the simulation period. They can be classified under four major headings: H-bonds, hydrophobic interactions, ionic interactions, and water bridges. The bars represent the % of the simulation time the specific interactions were maintained. Herein complex A (Fig. 20A) showed H-bonding with Val116 (100%), Arg120 (175%), Tyr355 (25%), Tyr385 (50%), and Ser530 (75%). As seen in docking studies, few of these H-bonds were conserved (Arg120). Moreover, curcumin was positioned in the active pocket so that it formed hydrophobic and water-bridged interactions with Val349, Phe205, Tyr348, Leu531, Ile89, Glu524, and His90. Complex B’s (Fig. 20B) position in the active site enabled it to form H-bonds with Tyr355 (70%), Tyr385 (70%), and Ser530 (60%). It also formed hydrophobic and water bridges with His90, Arg120, Val349, Phe518, Arg513, Glu524, and Leu534. As seen in docking studies, few of these H-bonds were conserved (Ser530 and Tyr355). Complex C (Fig. 20C) also positioned itself in the active site pocket and formed H-bonds with Ser171 (22%), Arg401 (22%), Ala606 (10%), and Leu615 (10%). It also formed hydrophobic and water bridges with Tyr181, Phe359, Leu368, Pro569, and Val433. The number of H-bonds formed was more than the internal ligand. All these observed interactions of curcumin were in accordance much better than the internal ligand’s interactions. Moreover, the number of H-bonds made by protein-curcumin complexes was almost more than those made by protein-internal ligand complexes. This shows the ability of curcumin to bind to active pocket site residues more effectively.

##### Interactions timeline

**Figure 21:**
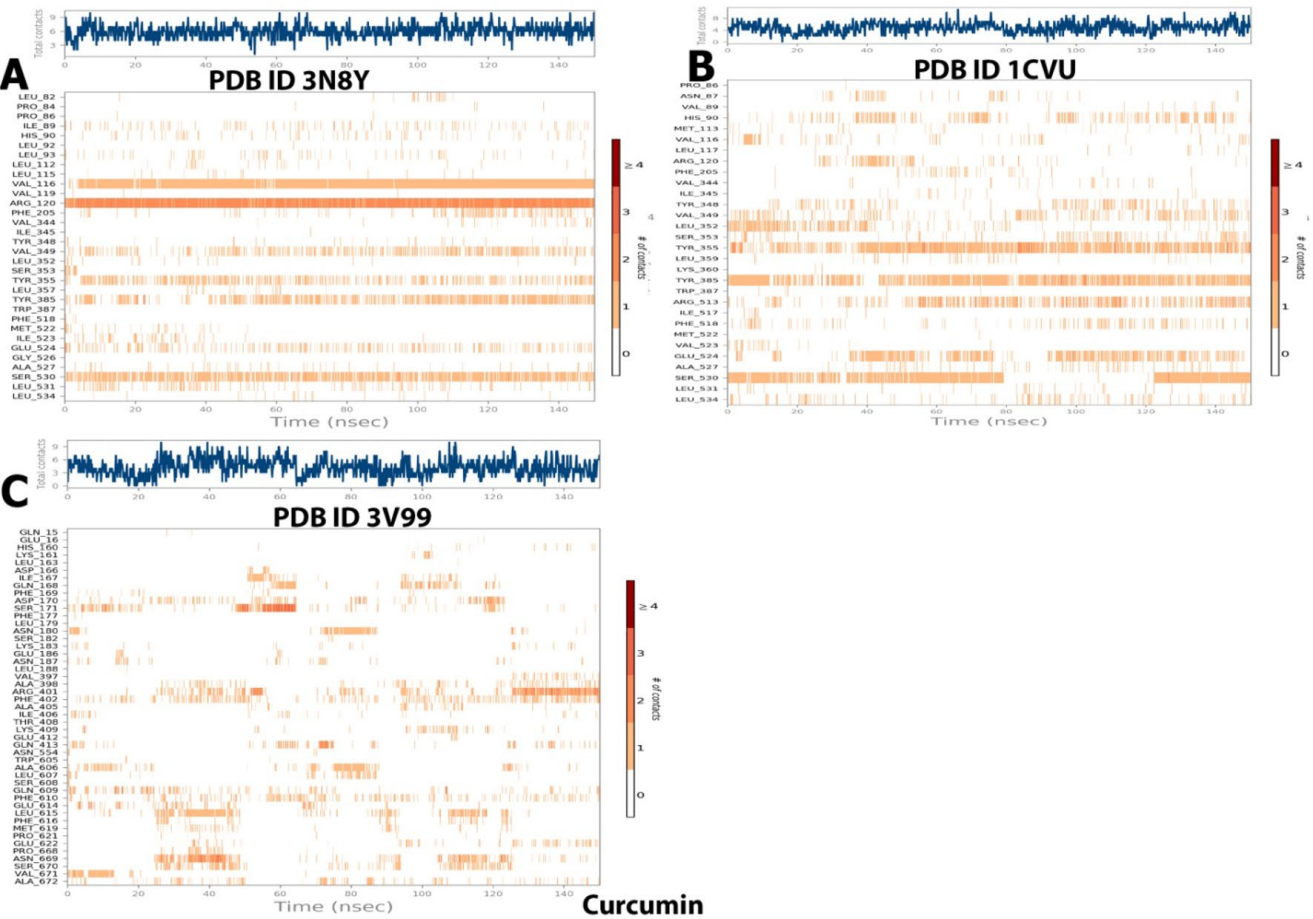
Specific contacts made by the proteins with the internal ligand throughout the simulation (dark color indicates more specific contact with the ligand) of (**A**) 3N8Y/curcumincomplex, (**B**) 1CVU/curcumin complex, and (**C**) 3V99/curcumin complex.

The interactions and contacts (H-bonds, hydrophobic, ionic, and water bridges) presented and shown in the form of a timeline in Fig. 21. The number of distinct interactions that the protein made with the ligand overall during the trajectory has been displayed in the top panel (dark blue). The residues that interact with the ligand in each trajectory frame are displayed in the bottom panel. According to the scale to the right of the plot, some residues have multiple specific contacts with the ligand, which is depicted by a darker orange color.

### MM-GBSA (binding free energy) calculations

The three ligands were subjected to a MM-GBSA analysis to determine the binding energies between the ligands and the proteins. It helps determine the protein–ligand complex’s stability after it is bound to the active site. Herein, the dG value represents the difference in energy between the optimized protein–ligand complex (prime) energy and a combination of optimized free ligand and protein energy. The detailed MM-GBSA dG binding values have been presented in Table 9.

**Table 9:** Tabular representation of MM-GBSA (dG) values (kcal/mol).

MM-GBSA of co-crystallized ligands							
Complex	dG binding	dG coulomb	dG covalent	dG H-bond	dG lipophilicity	dG solvation	dGvan der Waals
3V99-arachidonic acid	− 59.67	− 7.17	2.67	− 0.03	− 25.25	21.69	− 51.59
3N8Y- diclofenac	− 31.24	13.31	2.51	− 0.54	− 12.85	− 3.27	− 30.39
1CVU- arachidonic acid	1161.84	− 9.94	771.46	− 1.06	− 33.36	24.12	410.62
MM-GBSA of curcumin							
3V99-curcumin	− 27.72	− 3.24	1.35	− 1.01	− 15.77	27.74	− 36.78
3N8Y-curcumin	− 84.75	− 41.15	6.47	− 3.48	− 28.54	32.79	− 50.83
1CVU-curcumin	− 79.31	− 16.83	6.43	− 2.06	− 29.72	19.45	− 56.58

The MM-GBSA analysis revealed that curcumin had the best binding energy with all the three proteins under study (− 27.72 kcal/mol with 5-LOX, − 84.75 kcal/mol with COX-2, − 79.31 kcal/mol with COX-1). These binding energies were relatively better than the co-crystallized protein–ligand complex, highlighting curcumin’s potential in binding and inhibiting all three proteins under study.

### Density functional theory (DFT) calculation analysis

The DFT analysis investigated the binding affinity pattern of the ligands under investigation as it revealed electronic structural characteristics of the ligands. Using Koopman’s theorem, all three ligands were optimized to the B3LYP/6-311G level, and their molecular orbitals were investigated. Table 10 lists the various DFT parameters. The energy gap between the HOMO and LUMO was used to calculate ligand molecule’s stability and molecular reactivity, with a smaller energy gap corresponding to higher stability. The difference in bond energy clearly explains the active compound’s chemical reactivity and stability. The low [EHOMO-ELUMO] value indicates that the compounds are very reactive and stable. The chemical’s electronegativity (v) was negative, indicating that they interacted well with the protein molecule. Positive electronic chemical potential (l) values suggest that the compounds are chemically reactive.

**Table 10:** Various DFT calculations of ligands under study.

Ligand	Lumo	Homo	ΔE in eV	χ pauling	η in eV	σ	μ in eV	S	ω ev
Curcumin	− 0.02644652	− 0.2443655	0.217919006	− 0.13541	0.10896	9.177722	0.135406	4.588861	0.084136
Capsaicin	− 0.015558272	− 0.234630912	0.21907264	− 0.12509	0.109536	9.129392	0.125095	4.564696	0.071431
Gingerol	− 0.02433083	− 0.262221953	0.237891123	− 0.14328	0.118946	8.407207	0.143276	4.203604	0.086292

*LUMO* lowest unoccupied molecular orbital, *HOMO* highest occupied molecular orbital, *ΔE* energy gap (E_LUMO_—E_HOMO_), *χ* electronegativity, *η* chemical hardness, *S* chemical softness, *μ* chemical potential, *ω* electrophilic index.

**Figure 22:**
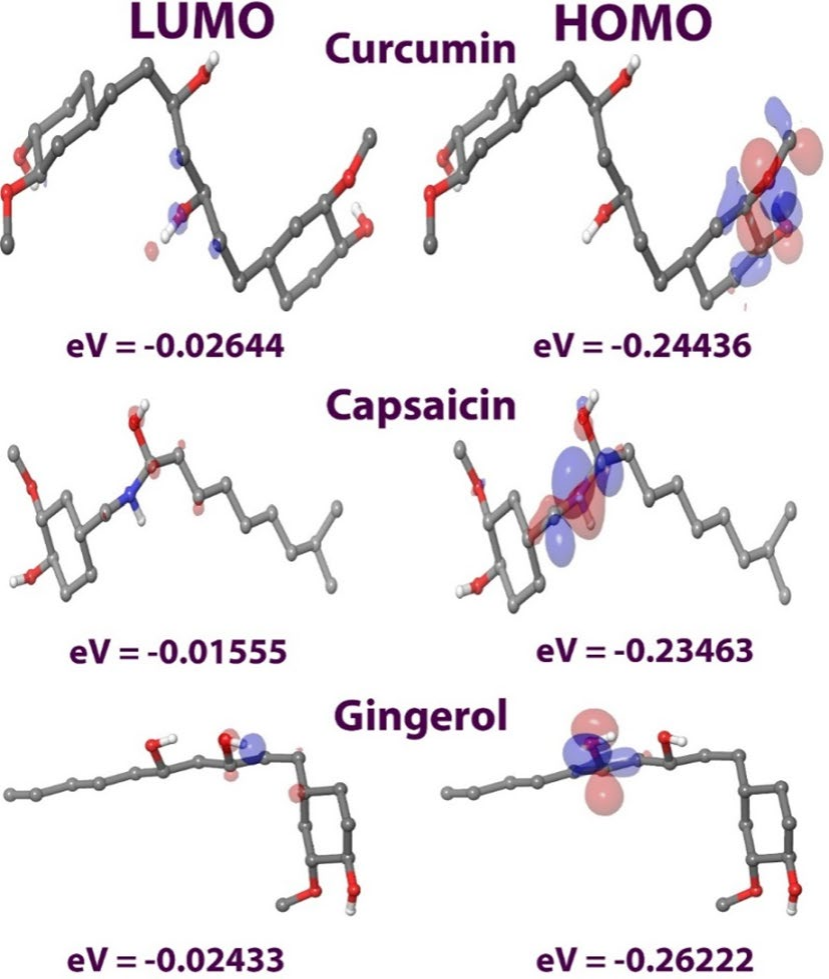
Pictorial representation of HOMO and LUMO energies for curcumin, capsaicin, and gingerol.

Furthermore, positive chemical hardness and chemical softness values enhance compound’s molecular interactions. Other metrics, such as polarizability (r) and electrophilicity (x), quantify the tendency to take an electron from its surroundings as a positive amount. Figure 22 depicts the HOMO–LUMO energy differential of the ligands using graphic diagrams. When the HOMO energy in a bioactive molecule is greater than the LUMO energy, the results can be assumed to be stable. The total DFT analysis indicates that the selected three ligands may exhibit stable behavior during interactions with all the proteins under study.

### Quantitative structure–activity relationship (QSAR) analysis

The QSAR analysis showed several critical aspects related to the chemical structures of the phytocompounds. Table 11 shows the QSAR rating of the phytocompounds. Surface area, volume, hydration energy, log P, refractivity, polarizability, mass, total energy, dipole moment, and RMS gradient are all investigated. Curcumin has the highest surface area and volume, followed by gingerol and capsaicin. Curcumin has the highest hydration energy, followed by capsaicin and gingerol. The highest Log P value was shown by capsaicin, followed by gingerol and curcumin. Curcumin has the highest refractivity value, followed by capsaicin and gingerol. Curcumin has the highest polarizability value, followed by capsaicin and gingerol. Curcumin, followed by capsaicin and gingerol, showed the highest mass. Curcumin showed the highest total energy, followed by capsaicin and gingerol. Gingerol showed the highest dipole moment, followed by capsaicin and curcumin. The highest RMS gradient was shown by curcumin, followed by capsaicin and gingerol.

**Table 11:** Tabular representation of QSAR rating of the ligands under study.

Property	Curcumin	Capsaicin	Gingerol
Surface area (Approx) (Å^2^)	636.52	671.01	602.09
Surface area (Grid) (Å^2^)	690.46	668.13	613.84
Volume (Å^3^)	1209.36	1104.86	1029.65
Hydration energy (Kcal/mole)	− 14.75	− 6.13	− 8.42
Log P	1.63	3.36	2.56
Refractivity (Å^3^)	104.37	90.79	84.64
Polarizability (Å^3^)	41.58	36.29	33.74
Mass (amu)	388.54	315.50	302.45
Total energy (kcal/mol)	33.4372	21.8707	19.8033
Dipole moment (Debye)	1.029	1.692	1.864
RMS gradient (kcal/ Å mol)	0.09391	0.0933	0.08983

### COX-1, COX-2 and 5-LOX inhibitory activities

Colorimetric enzyme immunoassay (EIA) was used to assess the inhibitory potential of the test compounds against COX-1, COX-2, and 5-LOX enzymes. The test concentration which produces 50% inhibition (IC_50_) was determined using serial dilution method (100, 10, 1, and 0.1 µM) for each compound. Ibuprofen, celecoxib, and zileuton were used as reference drugs for COX-1, COX-2, and 5-LOX, respectively. From results presented in Table 12, all the tested compounds (curcumin, gingerol and capsaicin) showed good potency towards inhibiting COX-1, COX-2, and 5-LOX enzymes.

**Table 12:** In vitro COX-1, COX-2 and 5-LOX inhibitory activity data.

Comp	IC_50_ data		
	COX-1 (µM)	COX-2 (µM)	5-LOX (µM)
Curcumin	18.58 ± 0.44	13.42 ± 0.92	24.08 ± 0.63
Capsaicin	24.42 ± 0.62	28.27 ± 0.55	36.44 ± 0.39
Gingerol	27.09 ± 0.82	32.82 ± 0.24	42.65 ± 0.17
Ibuprofen	6.52 ± 0.72	3.78 ± 0.29	–
Celecoxib	7.96 ± 0.41	0.62 ± 0.36	–
Zileuton	–	–	0.69 ± 0.72

*IC*_*50*_* (µM):* Concentration of test compound that produces 50% inhibition of COX-1, COX-2, and 5-LOX enzymes.

Data are expressed as mean three replicate observations ± SEM.

Ibuprofen, celecoxib and zileuton are reference standards.

– indicates not tested.

Among the three compounds, curcumin exhibited the best inhibitory activity against COX-1/2 and LOX enzymes. The inhibitory activities of capsaicin and gingerol were also prominent, but the extent of inhibition was less as compared to curcumin. When compared with the standard drugs, the IC_50_ values of the test compounds (curcumin, capsaicin, and gingerol) are comparable to some extent with ibuprofen and celecoxib (COX-I and COX-2 inhibitors) and zileuton (5-LOX inhibitor). However, curcumin possesses the best dual inhibitory activity towards COX-1/2 (IC_50_ = 18.58 ± 0.44 µM/13.42 ± 0.92 µM) and 5-LOX (IC_50_ = 24.08 ± 0.63 µM) enzymes. The in vitro inhibitory activities thus confirm the predicted inhibition of COX-1/2 and 5-LOX enzymes in molecular docking studies. Experimental investigation thus validates the in silico inhibition study.

## Discussion

The currently available therapeutics, i.e. NSAIDs that target various enzymes in the inflammation pathways, suffer from side effects. There is an urgent need to rediscover a single molecule with intense synergistic anti-inflammatory activity. This could only be possible through “dual inhibition of enzymes” in the inflammation pathway. In this perspective, we focused on exploring the role of culinary spice chemicals as dual inhibitors of key enzymes, COX and LOX in inflammatory pathways. There are strong evidences that several culinary spices could exert health benefits with strong antioxidant and anti-inflammatory properties.

Synergistic inhibition occurs when two or more bioactive compounds work together to enhance each other’s inhibitory effects, resulting in a greater overall effect than would be achieved by either compound alone. This can occur through a variety of mechanisms, including increased potency, increased bioavailability, and enhanced target specificity^40^. For example, several studies have shown that the combination of curcumin (major bioactive from turmeric) and piperine (major bioactive from black pepper) results in a synergistic inhibition of cancer cell growth. This is thought to occur because piperine enhances the bioavailability of curcumin, allowing it to more effectively target cancer cells. Another example is the combination of berberine (a natural product derived from plants) and metformin (a synthetic drug used to treat diabetes). It has been reported that the combination of these two compounds results in a greater reduction in blood sugar levels than either compound alone, suggesting a synergistic inhibition of diabetes. On this note, the concept of synergistic inhibition of natural products holds promise for the development of more effective and efficient therapies for a range of diseases and conditions^87^.This paves the way to employ the phytoconstituents of culinary spices as potent inhibitors of the enzymes involved in the inflammation pathways. Developing new and selective COX inhibitors requires knowledge of COX structure and isoform specificity. COX’s active site comprises a lengthy hydrophobic channel with a limited entry at the membrane-binding domain. Despite the fact that their active sites are quite similar, their binding cavities differ, with COX-2 having a bigger binding cavity than COX-1^88^. This created opportunities for the creation of specific COX inhibitors. The selectivity of COX inhibitors is determined by interactions with three distinct areas of the enzyme’s active site: the Arg120/Tyr355-containing entrance region, the hydrophobic pocket directly below the haem group, and the side pocket. Highly conserved sections include the active site entrance and the hydrophobic pocket; however, the side pocket is non-conserved, with a few different amino acid residues that give rise to an additional pocket in COX-2.COX-1 and COX-2 have highly similar active sites^89^ However, COX-2 has a side pocket just above the COX binding site’s entrance (Arg120 and Tyr355). The amino acids that comprise the 5-LOX binding site are primarily hydrophobic and form a deep-bent shape. Reports have indicated that Phe177 and Tyr181 in the cleft's top region form part of the 5-LOX binding site, with Trp599 and Leu420 at the cleft's base^90^. In addition, the polar amino acid Lys409 is present at the entrance of 5-LOX. The research suggested numerous other amino acid residues, including Tyr181, Leu414, Asn425, Arg411, and Phe421, are crucial for the interaction between 5-LOX and its substrate^67^. Hence, dual inhibition could be achieved by simultaneous inhibition of these two enzymes^11^.

As per our study, curcumin, the active ingredient of turmeric, displayed significant dual COX-1/2 and 5-LOX inhibitory activity. The results of molecular docking and dynamics studies can justify this. Hydrogen bond interactions with critical amino acids, such as Ser530 in the COX active site and *π-π* interactions between the ring B of the flavanone and Tyr355 at the entrance to the COX binding site are responsible for the COX-2 selectivity of curcumin. The hydrophobic contacts between the COX enzyme’s hydrophobic region and the lobby region further promoted effective binding. Curcumin’s expected activity in blocking 5-LOX comes from the hydrogen bond interactions withLys409, Ala672, and Phe177. This H-bonding was absent in the case of co-crystallized ligand. The hydrophobic and polar interactions were the primary contributors to the effective binding of curcumin. Molecular dynamics data further strengthened the results of molecular docking. Results revealed the high stability of curcumin-protein complexes despite minor acceptable deviations. Upon binding of curcumin in the protein’s active site, the complex displayed minor RMSD values. MMGBSA binding energies were quite better than the co-crystallized protein–ligand complex, highlighting curcumin’s potential in binding and inhibiting all the three proteins under study^55,59–68^. The other two spice chemicals, capsaicin and gingerol, displayed good binding modalities with the proteins under study. This study also explored the pharmacokinetics (ADMET) and the chemical interactions of the ligands under study, with various proteins and curcumin being the good among all. The present investigational data is also supported by DFT and QSAR studies.

There are some limitations regarding the analysis of natural products for inflammation associated with various diseases: (1) Complexity of natural products: Natural products are often complex mixtures of various compounds, which makes it difficult to isolate and identify the active compounds responsible for their anti-inflammatory effects. This complexity can also make it challenging to determine their mechanisms of action. (2) Lack of standardization: There is often a lack of standardization in the preparation and use of natural products, which can lead to variability in their potency and efficacy. This makes it difficult to compare results across different studies and to establish consistent dosing regimens. (3) Limited understanding of mechanisms of action: While natural products have been used for centuries to treat inflammatory conditions, the exact mechanisms by which they exert their anti-inflammatory effects are often poorly understood. This limits our ability to predict their efficacy in different disease contexts and to develop new drugs based on their mechanisms of action. (4) Limited clinical evidence: While there is a growing body of preclinical evidence supporting the anti-inflammatory effects of natural products, there is often a lack of well-designed clinical trials to support their use in humans. This makes it difficult to establish their safety and efficacy in clinical settings and to obtain regulatory approval for their use. (5) Interactions with other drugs: Natural products can interact with other drugs and medications, which can lead to unintended side effects or reduced efficacy. These interactions can be difficult to predict and may vary depending on the specific natural product and the individual patient.

Overall, while natural products show promise as potential treatments for inflammatory conditions, there are still many limitations to their use and further research is needed to fully understand their mechanisms of action and potential clinical applications.

## Conclusion

Our study pioneered investigating the role of culinary herbs as a potential inhibitor of the enzymes (COX and LOX) associated with inflammation. Herein our study identified a spice chemical, curcumin as a potential dual inhibitor of COX-1/2 and 5-LOX. This perspective identifies curcumin as a possible phytoconstituent of the COX-1/2 and 5-LOX dual pathway and offers it as potential drug inflammation-associated diseases. Overall, the curcumin has the potential for the dual inhibition of COX-2 and 5-LOX. In experimental inhibitory (in vitro) studies, curcumin exhibited the best dual inhibitory activities against COX-1/2 and 5-LOX enzymes. Capsaicin and gingerol also showed inhibitory potential against both COX and LOX enzymes. This study also highlights the important role of culinary herbs and Indian spices in inflammation associated with various disorders. In addition, our study also provides compelling support for the idea that consuming a diet rich in certain anti-inflammatory spices (curcumin, capsaicin and gingerol) can help reduce inflammation and protect against diseases associated with chronic inflammation. Although these spices have been used for millennia to alleviate inflammation, more research is needed to determine whether or if they may be used therapeutically to prevent or treat inflammatory conditions. In view of the anti-inflammatory potential these spice chemicals, this research could pave the way for more scientific exploration in this area for drug discovery.

## Data Availability

All data generated or analyzed during this study are included in this published article.
