# Author Correction: Dual synergistic inhibition of COX and LOX by potential chemicals from Indian daily spices investigated through detailed computational studies

**DOI:** 10.1038/s41598-024-66609-6

**Published:** 2024-07-16

**Authors:** Mithun Rudrapal, Wafa Ali Eltayeb, Gourav Rakshit, Amr Ahmed El-Arabey, Johra Khan, Sahar M. Aldosari, Bader Alshehri, Mohnad Abdalla

**Affiliations:** 1 Department of Pharmaceutical Sciences, School of Biotechnology and Pharmaceutical Sciences, Vignan’s Foundation for Science, Technology & Research (Deemed to Be University), Guntur, 522213 India; 2 Biotechnology Department, Faculty of Science and Technology, Shendi University, Shendi, 414601 Sudan; 3 Department of Pharmaceutical Sciences and Technology, Birla Institute of Technology, Ranchi, 835215 India; 4 Department of Pharmacology and Toxicology, Faculty of Pharmacy, Al-Azhar University, Cairo, 11651 Egypt; 5 Department of Medical Laboratory Sciences, College of Applied Medical Sciences, Majmaah University, 11952 Al’Majmaah, Saudi Arabia; 6 Health and Basic Sciences Research Center, Majmaah University, 11952 Al’Majmaah, Saudi Arabia; 7 Pediatric Research Institute, Children’s Hospital Affiliated to Shandong University, Jinan, 250022 People’s Republic of China

Correction to: *Scientific Reports* 10.1038/s41598-023-35161-0, published online 27 May 2023

The original version of this Article contained an error in the Acknowledgements section.

“The authors thank the Deanship of Scientific Research at Majmaah University, Saudi Arabia, for supporting this research work under project number (IFP-2023-445).”

now reads:

“The authors thank the Deanship of Scientific Research at Majmaah University, Saudi Arabia, for supporting this research work under project number (R-2023-445).”

The original Article has been corrected.

